# Implications of metabolism on multi‐systems healthy aging across the lifespan

**DOI:** 10.1111/acel.14090

**Published:** 2024-01-29

**Authors:** Shanshan Yao, Laura A. Colangelo, Andrew S. Perry, Megan M. Marron, Kristine Yaffe, Sanaz Sedaghat, Joao A. C. Lima, Qu Tian, Clary B. Clish, Anne B. Newman, Ravi V. Shah, Venkatesh L. Murthy

**Affiliations:** ^1^ University of Pittsburg Pittsburgh Pennsylvania USA; ^2^ Northwestern University Chicago Illinois USA; ^3^ Vanderbilt University Medical Center Nashville Tennessee USA; ^4^ University of California San Francisco California USA; ^5^ University of Minnesota Minneapolis Minnesota USA; ^6^ Johns Hopkins University Baltimore Maryland USA; ^7^ National Institute of Aging Baltimore Maryland USA; ^8^ Broad Institute of Harvard and MIT Cambridge Massachusetts USA; ^9^ University of Michigan Ann Arbor Michigan USA

**Keywords:** aging, metabolomics, mechanisms

## Abstract

Aging is increasingly thought to involve dysregulation of metabolism in multiple organ systems that culminate in decreased functional capacity and morbidity. Here, we seek to understand complex interactions among metabolism, aging, and systems‐wide phenotypes across the lifespan. Among 2469 adults (mean age 74.7 years; 38% Black) in the Health, Aging and Body Composition study we identified metabolic cross‐sectionally correlates across 20 multi‐dimensional aging‐related phenotypes spanning seven domains. We used LASSO‐PCA and bioinformatic techniques to summarize metabolome‐phenome relationships and derive metabolic scores, which were subsequently linked to healthy aging, mortality, and incident outcomes (cardiovascular disease, disability, dementia, and cancer) over 9 years. To clarify the relationship of metabolism in early adulthood to aging, we tested association of these metabolic scores with aging phenotypes/outcomes in 2320 participants (mean age 32.1, 44% Black) of the Coronary Artery Risk Development in Young Adults (CARDIA) study. We observed significant overlap in metabolic correlates across the seven aging domains, specifying pathways of mitochondrial/cellular energetics, host‐commensal metabolism, inflammation, and oxidative stress. Across four metabolic scores (body composition, mental‐physical performance, muscle strength, and physical activity), we found strong associations with healthy aging and incident outcomes, robust to adjustment for risk factors. Metabolic scores for participants four decades younger in CARDIA were related to incident cardiovascular, metabolic, and neurocognitive performance, as well as long‐term cardiovascular disease and mortality over three decades. Conserved metabolic states are strongly related to domain‐specific aging and outcomes over the life‐course relevant to energetics, host‐commensal interactions, and mechanisms of innate immunity.

## Graphical abstract

Metabolic signatures across aging phenome in an older population are consistently and independently related to domain‐specific aging and long‐term CVD and mortality risk in younger individuals. Conserved metabolic states are strongly related to domain‐specific aging and outcomes over the life course relevant to energetics, host‐commensal interactions, and mechanisms of innate immunity.

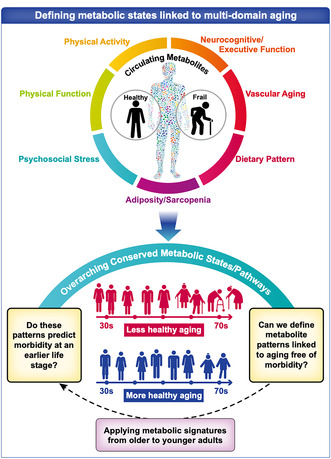

**Abbreviations**
BMI: body mass index
CARDIA: coronary artery risk development in young adults
CV: coefficient of variation
CVD: cardiovascular disease
FDR: false discovery rate
Health ABC: health, aging and body composition study
HMDB: human metoblome database
KEGG: kyoto encyclopedia of genes and genomes
LASSO: least absolute shrinkage and selection operator
LC‐MS: liquid chromatography‐mass spectrometry
PC: principal component
PCA: principal component analysis

## 1 INTRODUCTION

Nearly 1 in 2 Americans between age 75–84 years old cite at least one limiting disability (He & Larsen, 2014), driving efforts to understand how best to compress this morbidity to enable healthier living into older age. Most efforts to understand “healthy aging” have relied on the epidemiology of biochemical and phenotypic frailty measures in individuals with advanced age, relying on collection of in‐person phenotyping at an advanced stage of aging that may not be specific to underlying, targetable metabolic mechanisms. Indeed, the recognition of altered metabolism and inflammation in age‐related morbidity (Murthy et al., 2016) has prompted recent approaches using biomarkers of metabolic dysfunction (e.g., inflammation, oxidative stress, muscle/endocrine function) to augment phenotype‐based approaches (Banerjee et al., 2011; Burkle et al., 2015; Gruenewald et al., 2006; Stenholm et al., 2010; Walston et al., 2006), ushering a renaissance of broader “omic” discovery efforts (Cheng et al., 2015; Ho et al., 2016; Lewis et al., 2010; Liu et al., 2022; Murphy et al., 2017; Walford et al., 2016; Wang et al., 2013). For instance, metabolic studies focused on longevity (Cheng et al., 2015) have specified metabolites relevant to energetics (e.g., isocitrate, aconitate) related to specific age‐related cardiac outcomes (e.g., heart disease and cancer), with replication in model systems of aging (citric acid cycle metabolites in *Caenorhabditis elegans*; Hamilton et al., 2005). While the molecular epidemiology of aging has understandably focused on functional biomarkers and phenotypes in older individuals, metabolic and epigenetic alterations occurring earlier in life may impact or accelerate the aging process. Uncovering the metabolic architecture of how humans age in a “healthy” manner across the life‐course—specifically whether pathways of morbidity compression in older age are relevant at an earlier age—is a critical step in prioritizing pathways and mechanisms of risk in aging biology.

Here, we harness deep phenotyping and surveillance in 2469 participants of the Health, Aging and Body Composition study (Health ABC; age 70–79 at study entry) alongside broad metabolite profiling to identify a potential metabolic underpinning of age‐related disease. Using statistical learning techniques across 20 aging phenotypes spanning multiple organ systems (physical, vascular, neurologic, and adipose), we identified four metabolic signatures reflecting physical activity, mental‐physical function, body composition, and vascular function. We subsequently tested those signatures against long‐term outcomes (including aging free of comorbidity or disease) in Health ABC and in a separate cohort of >2000 young adults in the Coronary Artery Risk Development in Young Adults study (CARDIA) with >2 decades of follow‐up from the fourth decade of life forward for outcomes, specifically similar conditions reflecting age‐related morbidity. Our ultimate goal was to understand the impact of metabolic health on specific systems‐wide phenotypes relevant to aging and its implication on early development of these phenotypes and compression of morbidity in later adulthood.

## 2 METHODS

### 2.1 Study populations

We studied two independent cohorts of individuals with metabolite profiling in this study: (1) participants from the Health, Aging and Body Composition study (Health ABC; *N* = 2469); (2) the Coronary Artery Risk Development in Young Adults study (CARDIA; *N* = 2320).

Health ABC is a prospective cohort of 3075 Black and White older adults aged 70–79 recruited from Pittsburgh, Pennsylvania, and Memphis, Tennessee during March 1997 to July 1998 (Visser et al., 2005). Participants were selected to have limited age‐related comorbidity, including ability to walking at least 1/4 mile, climb up 10 steps, and conduct basic activities of daily living. In addition, participants could not have a cancer treatment in the past 3 years and or plans to leave the study area in the following 3 years. We assessed metabolomics in blood samples from the 1998 to 1999 (Year 2) examination. Covariates included age, sex, race, highest education level, smoking status, and body mass index (BMI). Baseline history or presence of hypertension and diabetes were based on self‐report of a physician diagnosis or taking medication for the disease. Participants brought all prescription medications used in the last 2 weeks for the medication inventory. Defining measures of multi‐systems aging (described in Table 1; Table S1) spanned 20 healthy aging phenotypes in 7 domains: physical function, neurocognitive/executive function, adiposity/sarcopenia, vascular, dietary pattern, physical activity, and psychosocial stress. Our primary outcome in Health ABC was “exceptionally healthy aging” defined by avoidance of a multi‐parametric outcome of a composite major disability, dementia, cancer, CVD, or all‐cause mortality over 9 years. The secondary outcomes were the five components of “exceptionally healthy aging” modeled separately.

**TABLE 1:** Measurement of aging phenome and incident outcomes. References are provided to denote specific cohort reports and definitions for clarification.

Aging phenome	Measurements	
Phenome domains	Health ABC	CARDIA
Physical function	Year 2 20 m gait speed (m/s) (Atkinson et al., 2007) Baseline Epidemiologic Studies of the Elderly (EPESE) battery (0–12) (Brach et al., 2004; Simonsick et al., 2001) Baseline Health ABC performance battery (0–4) (Brach et al., 2004; Simonsick et al., 2001)	Exercise treadmill time (min) (Baseline and Year 20)
Physical activity	Year 2 weekly walk time (min) Year 2 energy expenditure (kcal/kg/week)	Questionnaire‐based physical activity (activity units) (baseline and Year 25)
Neurocognitive/executive function	Baseline Teng‐modified Mini‐Mental Status (Teng's 3MS) Exam (0–100) (Rosano et al., 2005) Baseline digit symbol substitution (DSST) test (Rosano et al., 2005) Year 3 scored clock drawing task (CLOX1) (0–15) (Atkinson et al., 2007) Year 3 15‐item Executive Interview (EXIT15) (0–30) (Atkinson et al., 2007)	Rey Auditory Verbal Learning Test (RAVLT), including long‐delay recall (Year 25, 30) Stroop Test (interference score) (Year 25, 30) Digit Symbol Substitution Test (DSST, Year 25, 30) Calculated Letter and Category Fluency (Year 30) Montreal Cognitive Assessment (MOCA, Year 30)
Adiposity/sarcopenia	Baseline thigh intermuscular fat area (cm^2^) (Abbatecola et al., 2012; Beavers et al., 2013; Huynh et al., 2022; Madero et al., 2017) Baseline total quadriceps muscle area (cm^2^) (Abbatecola et al., 2012; Beavers et al., 2013; Huynh et al., 2022; Madero et al., 2017) Baseline abdomen visceral fat area (cm^2^) (Abbatecola et al., 2012; Beavers et al., 2013; Huynh et al., 2022; Madero et al., 2017) Baseline abdomen subcutaneous fat area (cm^2^) (Abbatecola et al., 2012; Beavers et al., 2013; Huynh et al., 2022; Madero et al., 2017) Baseline abdomen visceral‐subcutaneous (Visceral: SubQ) fat area ratio Baseline average thigh muscle density (Hounsfield units) (Abbatecola et al., 2012; Beavers et al., 2013; Huynh et al., 2022; Madero et al., 2017) Year 2 isokinetic strength (N‐m) (Brach et al., 2004)	Body mass index (kg/m^2^) (Baseline, Year 25) Pericardial fat volume (mm) (Year 25) Subcutaneous fat volume (cm^3^/10 mm) (Year 25) Average psoas muscle volume (cm^3^) (Year 25) Visceral and total abdominal fat volume (cm^3^/10 mm) (Year 25)
Vascular aging	Baseline arterial pulse wave velocity (PWV) (cm/s) (Abbatecola et al., 2012)	Cardiovascular risk factors (Year 7) Coronary and aortic calcification (Agatston score) (Year 25) Left ventricular mass index (g/m^2.7^) (Year 25, 30)
Dietary pattern	Year 2 Healthy Eating Index (HEI) score (0–100) (Lee et al., 2004) Year 2 daily glycemic load (Lee et al., 2004)	Life Simple Seven score (American Heart Association, Baseline)
Psychosocial stress	Baseline HABC psychosocial risk index (0–4) (Vogelzangs et al., 2007)	‐
Outcomes		
Exceptionally healthy aging	Surviving to Year 10 visit without CVD, cancer, dementia, or major disability	All‐cause mortality
CVD	Incident myocardial infarction, angina, heart failure and/or stroke	Incident CVD including myocardial infarction, non‐MI acute coronary syndrome, stroke, heart failure, carotid artery disease, peripheral arterial disease, atherosclerotic disease, and non‐atherosclerotic cardiac disease
Cancer	Incident cancer, except non‐melanoma skin cancer	Incident cancer, except non‐melanoma skin cancer
Dementia	Incident dementia (medical records, medication, and cognition tests; Aubert et al., 2017)	Continuous metrics above
Major disability	Needing equipment, having mobility disability or needing help with an activity of daily living	Continuous metrics above
All‐cause mortality	Death of all causes	All‐cause mortality

The CARDIA cohort is an ongoing longitudinal investigation of Black and White participants aged 18–30 years at recruitment across four American centers (Friedman et al., 1988). The Year 0 visit was conducted in Year 1985–1986. We studied 2320 individuals with metabolite profiling with complete covariate data (see Section 2.3) at their Year 7 study visit (Year 1992–1993; in a reported 8‐h fasting state), as described (Murthy et al., 2022; Shah et al., 2023). CARDIA participants have been longitudinally followed with repeated assessments for a variety of exposures and outcomes, including anthropometry, cardiovascular risk factors, and incident cancer, cardiovascular disease, and mortality (Friedman et al., 1988). Given CARDIA is by design a substantially younger population relative to Health ABC, we selected outcomes in CARDIA to harmonize with several age‐related domains (Table 1), including neurocognitive function, cardiovascular phenotypes, adiposity and muscle phenotypes, physical activity and fitness, and dietary patterns in Health ABC. In addition, we included a composite measure of ideal cardiovascular health (American Heart Association Life's Simple Seven score) (Ruiz‐Ramie et al., 2021). Methods for adjudication of each of the endpoints in CARDIA are provided in references in Table 1. In brief, physical activity was quantified via questionnaire (Jacobs Jr. et al., 1989), and methods for adjudication of computed tomographic indices of adiposity and muscle quantity (Granados et al., 2019; Oh et al., 2022), fitness (Shah et al., 2022), cardiac phenotypes (Shah et al., 2016), and neurocognitive assessments (Reis et al., 2013) have been previously described.

The institutional review boards of participating institutions of both studies approved the study, and all participants provided written informed consent.

### 2.2 Metabolite profiling

Circulating metabolites in Health ABC were measured in fasting plasma samples (≥8 h; no prior thaw) collected at Year 2 visit using liquid chromatography‐mass spectrometry (LC‐MS) methods at the Broad Institute (Cambridge, MA) as described in our previous work (Murthy et al., 2022; Shah et al., 2023). Identical methods were utilized for measurement of metabolite profiles in CARDIA although the samples were run separately at the Broad Institute, as described (Murthy et al., 2022; Shah et al., 2023). Among the 520 identified metabolites in samples from both cohorts, 478 were measured in 90% of the participants in the Health ABC samples and with a coefficient of variation (CV) ≤10% and were included in analyses. Metabolites quantified in more than one platform were included in analyses. The metabolite values for each cohort were separately processed for further analyses. Missing values for metabolites were imputed as 50% of the lowest value detected among all original samples in each cohort separately. Metabolites were log transformed, mean‐centered, and standardized for regression. Sensitivity analyses for potential batch effects within Health ABC samples during the COVID‐19 pandemic are documented in Data S1.

### 2.3 Statistical analysis

The study scheme is shown in Figure S1. Broadly, we (1) developed metabolic signatures of aging phenotypes in Health ABC; (2) tested their associations with aging‐related outcomes in Health ABC; and (3) examined their associations with aging phenotypes and outcomes at an earlier stage of life in CARDIA.

#### 2.3.1 Discovery cohort (Health ABC)

Distributions of continuous subclinical phenotypes were graphically examined, with transformation as appropriate to improve normality (e.g., log). Thigh intermuscular fat area, thigh quadriceps muscle area, abdomen visceral fat area, and abdomen subcutaneous fat area were indexed to height^1.7^ based on prior literature and dependence on height to limit dependence on body size (Brown et al., 2022; Heymsfield et al., 2011). Phenotypes were mean‐centered and standardized for regression. Of note, to facilitate interpretation, select phenotypes (EXIT‐15, thigh intermuscular fat area, abdomen visceral fat area, abdomen subcutaneous fat area, visceral‐subcutaneous fat area ratio, PWV, daily glycemic load, and Psychosocial Risk Index) were signed so that a higher numeric value for all phenotypes reflected a “healthier” measure. We subsequently fit age, sex, and race‐adjusted linear models for each of the 20 aging phenotypes as a function of each metabolite with a false‐discovery rate correction (Benjamini‐Hochberg) for multiplicity.

To generate parsimonious models of composite aging phenotypes (across all 20 phenotypes), we employed LASSO regression and principal components analysis (PCA) methods to construct signatures across all phenotypes as detailed in Data S1 (Murthy et al., 2020). Of note, to further increase parsimony, prior to the LASSO regression, we used a variable pruning method (*findCorrelation* in *caret* in R) to select metabolites among those also measured in CARDIA (*n* = 478, to facilitate validation) for entry into LASSO, which considered pair‐wise correlations greater than 0.8 across all metabolites and iterated removal of the metabolite with the largest mean absolute mean correlation with others until all metabolites were with <0.8 absolute correlation with others. We then used LASSO regression based on the selected metabolites (*n* = 318) to select an explanatory model for each of the 20 phenotypes (phenotype as dependent variable and the entire metabolome included as independent variables for LASSO selection). We next employed a PCA approach using coefficients derived from LASSO regressions. Unlike conventional PCA, which is performed on the values of metabolites from individual subjects, here we performed PCA on the regression coefficients from LASSO models, as markers of the strength of the relation between all metabolites and all phenotypes, across all 20 phenotypes. This approach generates principal components (or aging “axes”) of covariation between the metabolome and aging phenome that enables construction of parsimonious metabolite signatures for each aging axis. We selected the number of resulting components (PCs) by a scree plot (in this case, this yielded four PCs; see Figure S2 and Section 3) and a ≥10% proportion of variance being explained by each PC. Each metabolite was assigned a weight (PC score) for each PC (that captures the strength of the relation of the metabolite to the PC), and a linear combination of metabolite weights (PC scores) and the circulating metabolite level was used to generate the metabolic score for each study participant reflective of each PC phenotype domain. Finally, we measured the associations of these standardized metabolic scores with incident clinical outcomes (exceptionally healthy aging and its components) after Year 2 visit using Cox regression (serially adjusted for age, sex, race, baseline education, baseline smoking status, BMI, baseline hypertension, diabetes, and baseline renal function by cystatin C). Given that we examined mortality and a composite “exceptionally healthy aging” as primary endpoints, we did not conduct further competing risk analysis in Cox models.

#### 2.3.2 Early adulthood extension cohort (CARDIA)

We applied metabolite weights (PC scores) from PCA developed in Health ABC to generate analogous metabolic scores of each aging component in CARDIA and tested their relation to sex and race. In addition, we used these scores in linear models for each outcome (Table 1), adjusted in initial models for age, sex, and race, and further in fully adjusted models for education, income, cigarettes per day and lifetime smoking (until Year 7), hypertension, BMI, total and high‐density lipoprotein cholesterol, and diabetes at Year 7 (the time of the metabolite measures). Of note, models for Year 20 exercise treadmill time was adjusted for Year 7 (baseline) treadmill time, and models for Year 7 Life's Simple Seven score (Lloyd‐Jones et al., 2010) was adjusted only for age, sex, race, education and income (given the incorporation of other metrics within the Life's Simple Seven score). Cox models were used to estimate the associations between metabolic scores and incident cancer (defined as any self‐reported cancer excluding non‐melanoma skin cancer), cardiovascular disease, and all‐cause mortality, with similar adjustments as in linear models. The Year 7 examination was taken to be the time origin for the survival analysis using Cox regression analyses.

### 2.4 Pathway analysis

Pathway analyses were conducted to identify enriched pathways for metabolites associated with each phenotype (5% false discovery rate) in unpenalized linear models. MetaboAnalystR package (Chong & Xia, 2018) was utilized to map Human Metabolome Database (HMDB) identification numbers (IDs) of the metabolites to Kyoto Encyclopedia of Genes and Genomes (KEGG) IDs. Subsequently, KEGG IDs were mapped to genes based on MetaboAnalyst's internal databases (Chong et al., 2018), which were generated from information gathered primarily from HMDB and search tool for interactions of chemicals (STITCH) databases (Yao et al., 2015). To ensure mapping specificity from metabolites to genes, only the top 50 genes with the highest mapping score for each metabolite were considered. Pathway enrichment analyses were then performed on gene sets associated with metabolites in each phenotype respectively. All metabolites measured in this study were mapped to genes with the same criteria and used as background genes for enrichment analysis. For result visualization, the top three most enriched pathways in each phenotype were selected and visualized together in a heatmap.

## 3 RESULTS

### 3.1 Clinical characteristics

Clinical characteristics of both cohorts included in our study are shown in Table 2. Health ABC participants were older at time of metabolomic profiling (74.7 ± 2.9 years), with an even distribution by sex (50% women) and 38% were Black. In this older cohort, the prevalence of hypertension and diabetes was higher than in CARDIA (50% vs. 14% and 12% vs. 2%, respectively). By design, CARDIA participants were younger (32.1 ± 3.6 years) with a similar sex and race distribution and less prevalent cardiometabolic comorbidity. The distribution of the 20 aging phenotypes included in our study in Health ABC and in CARDIA are displayed in Tables S1 and S2.

**TABLE 2:** Characteristics of study participants from the Health ABC and CARDIA studies. CARDIA study characteristics provided as available. Missing <10% of covariates in both studies.

Characteristic	Health ABC (*N* = 2,469)	CARDIA (*N* = 2320)
Age, years	74.7 (2.9)	32.1 (3.6)
Black	934 (38%)	1032 (44%)
Women	1,233 (50%)	1036 (45%)
More than high school education	1,879 (76%)	1721 (74%)
Current smoker	227 (9%)	540 (23%)
Body mass index, kg/m^2^	27.2 (4.8)	25.7 (5.0)
Prevalent conditions		
Cardiovascular disease	652 (27%)	125 (5%)
Hypertension	1,232 (50%)	279 (12%)
Diabetes	357 (14%)	50 (2%)
Cancer	486 (20%)	195 (8%)
Peripheral artery disease	124 (5%)	‐
Osteoarthritis	242 (10%)	‐
Depression	228 (9%)	‐
Pulmonary disease	283 (12%)	‐
Total number of medications (medication inventory)	3.2 (2.7)	‐

*Note*: Values are expressed as mean (standard deviation) or *n* (%). Cancer cases before 4 years of follow‐up after metabolite profiling in CARDIA were considered “prevalent” and excluded from Cox models.

### 3.2 Metabolites associated with aging phenotypes define conserved and novel pathways relevant to multi‐organ healthy aging

**FIGURE 1:**
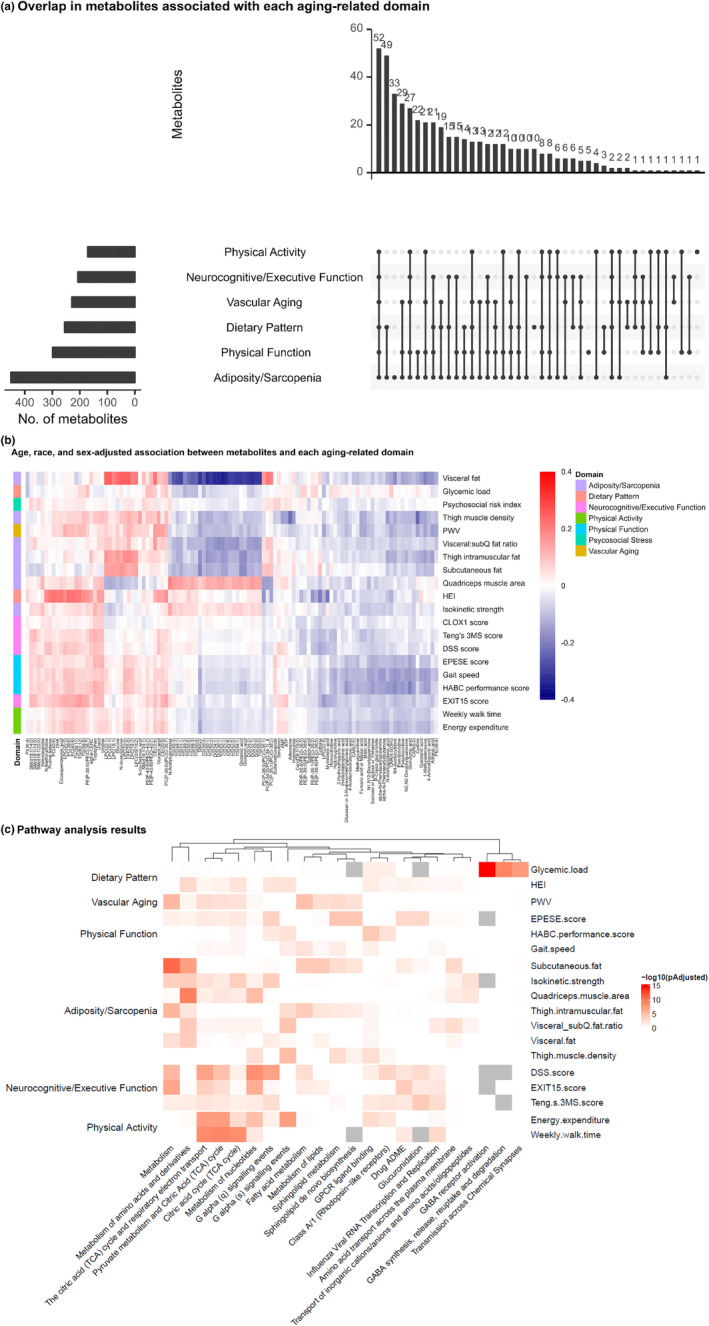
Metabolic architecture of multi‐domain aging. (a) Shows overlap in metabolites associated with each aging‐related domain in Health ABC (in age, sex, race‐adjusted regression). The bar graph to the left of the aging domains specifies the total number of metabolite associations (at a 5% false discovery rate). We did not observe any significant metabolite associations with psychosocial stress (not shown). (b) Displays the age, race, and sex‐adjusted regression estimates for top 20 metabolites (by magnitude) in association with aging phenotypes across aging domains in Health ABC (phenotypes in columns; metabolites in rows). Positive coefficients indicated a greater metabolite level was associated with a prognostically better value of the phenotype. (c) Represents results of pathway analysis (as described in Section 2). Two phenotypes (CLOX1 score, Psychosocial risk index) did not have any metabolites significant at 5% false discovery rate and are not listed. Grey box indicates there was no gene implicated in the given pathway by the metabolomic associations.

To define metabolic pathways of healthy aging, we first identified metabolites associated with each of the 20 included phenotypes across seven pre‐specified aging‐related domains (physical function, neurocognitive/executive function, adiposity/sarcopenia, vascular aging, dietary pattern, psychosocial stress, and physical activity) in age‐, sex‐, and race‐adjusted linear models (full regression estimates in Data S1; Figure 1a,b). We observed general concordance of metabolite associations across phenotypic domains (except for psychosocial stress, which did not have any metabolite associations that survived type 1 error correction). In total, 485 metabolites were related to at least one of the aging phenotypes, where the greatest proportion of them, *n* = 52 (11%), were shared across the six aging domains. Select metabolites with significant associations across multiple domains are shown in Table 3. Although our results identified metabolites well‐established in the molecular pathophysiology of longevity (cis‐aconitic acid (Cheng et al., 2013; Cheng et al., 2015; Yan et al., 1997; Yarian et al., 2006), isocitrate (Cheng et al., 2015)), a principal theme was identification of metabolites central to canonical and emerging integrated pathways of oxidative stress, inflammation, and central metabolic control (e.g., AMP kinase/mTOR regulation, mitochondrial substrate availability, and function; e.g., hydrocinnamic acid (Alam et al., 2016; Ong et al., 2012; Sakai et al., 1997; Taofiq et al., 2017), lysophosphatidylcholines (Semba et al., 2019), 2‐aminobutyric acid (Irino et al., 2016), Imidazole propionic acid (Koh et al., 2018; Molinaro et al., 2020), carnitines, leukotriene pathway metabolites; see Table 3). Importantly, several metabolites had been implicated in inflammation broadly, but not clearly in human aging: for example, adrenic acid—an omega‐6 fatty acid—has been demonstrated to reduce endoplasmic reticular stress (Singh et al., 2021) and may act in inflammation resolution via modulation of inflammatory cell function (Brouwers et al., 2020). Finally, we noted that several metabolites were microbially derived and potentially modifiable with lifestyle: for example, indole‐3 propionic acid (a microbial product of tryptophan metabolism) may have broad effects on multiple aging organ systems (e.g., neurologic, pro‐inflammatory adiposity, CVD (Bendheim et al., 2002; Serger et al., 2022; Xue et al., 2022; Zhao et al., 2019)) and responds to Mediterranean (but not Western) diet (Zhu et al., 2020). While a full exposition of metabolites implicated across all aging phenotypes is difficult in the scope of our work, unbiased analysis of pathways implicated by metabolites across phenotypes has suggested key shared pathways across multiple phenotypes, including one‐carbon and amino acid metabolism and lipid and fatty acid metabolism, among others (Figure 1c).

**TABLE 3:** Selected known and novel metabolites with implications in multi‐domain aging phenotypes.

Metabolite	Psychosocial Stress	Dietary pattern	Neurocognitive/Executive Function	Physical activity	Physical function	Adiposity/sarcopenia	Vascular aging	Clinical–pathologic associations
Hydrocinnamic acid	↑↑	↑	↑	↑↑	↑↑	↑↑	↑↑	Master class of phenolic acid natural products from fruits/vegetables (Alam et al., 2016); anti‐inflammatory, anti‐oxidant roles with roles in multi‐organ function (adipose, liver, CVD, and renal) (Alam et al., 2016; Taofiq et al., 2017); blunts innate inflammatory response to LPS (Sakai et al., 1997) and inhibits NF‐kB activation (Jung, Go, Kim, Yu, & Chung, 2009); metabolic activation of AMP kinase (Ong et al., 2012)
Lysophosphatidylcholines (LPCs)	↑	‐	‐	↑	↑↑	↑↑	↑↑	Several species linked to decreased mitochondrial oxidative capacity in humans (Semba et al., 2019); related to hepatic and endothelial function, oxidative stress (pro‐inflammatory) (Law et al., 2019)
2‐aminobutyric acid	↑↑	↑↑	↑↑	↑↑	↑↑	‐	↑↑	Potential role in regulation of glutathione pools; can increase intracellular glutathione via AMPK activation (Irino et al., 2016)
3‐indolepropionic acid (indole‐3‐propionic acid)	↑	↑↑	↑↑	↑↑	↑↑	↑↑	↑↑	Gut microbial product of Trp metabolism; broad effects on neurocognitive disease and nerve growth (Bendheim et al., 2002; Serger et al., 2022), CVD (Xue et al., 2022), hepatic steatosis (Zhao et al., 2019); increases with Mediterranean (but not Western) diets (Zhu et al., 2020); potential metabolic effects via modulation of GLP‐1 release in intestine (Chimerel et al., 2014)
Fumaric (or maleic) acid	↓↓	↓↓	↓↓	↓↓	↓↓	↓↓	↓↓	Fumaric acid esters shown to reduce inflammation and oxidative stress in spontaneously hypertensive rats (including effects in multiple organs: heart, kidney) (Silhavy et al., 2014); may extend lifespan in *C. elegans* (Edwards, Copes, Brito, Canfield, & Bradshaw, 2013)
Glyceric acid	↓	↑↑	↑↑	↑↑	↑↑	↑↑	↑↑	May promote improved mitochondrial function in older humans (Hirvonen, Kyrolainen, Lehti, & Kainulainen, 2021)
Kynurenic acid, quinolinic acid	↑	↑↑	↓	↓↓	↓↓	↓↓	↓↓	Kynurenine pathway metabolites; quinolinic acid levels in plasma and CSF associated with age (Sorgdrager et al., 2019), and may modulate neurological function via NMDA receptor stimulation (Lugo‐Huitron et al., 2013); may be related to cognitive deficits (Cathomas, Guetter, Seifritz, Klaus, & Kaiser, 2021)
Alpha‐N‐phenylacetylglutamine	↓↓	↓	↓↓	↓↓	↓↓	↓↓	↓↓	Gut microbial product (glutamine and phenylacetate); related to heart failure (Romano et al., 2023), CVD (Ottosson et al., 2020), stroke (F. Yu et al., 2021), and thrombosis (Nemet et al., 2020); mechanism may involve adrenergic receptor signaling (Nemet et al., 2020)
Carnitines	‐	↓	↓	↓↓	↓↓	↓↓	↓↓	Involved in mitochondrial beta‐oxidative capacity
Long chain fatty acids, EPA^a^ (and unsaturated triglycerides)	↑	↑↑	↑↑	↑↑	↑↑	↑	↑↑	Examples include myristoleic acid and multiple unsaturated triglycerides; found in dietary sources (e.g., fish) (Mazzilli et al., 2020); EPA has been associated with lower CVD risk and healthy aging free of morbidity (Lai et al., 2018)
*cis*‐aconitic acid	↓	↓↓	↓↓	↓↓	↓↓	↓↓	↓↓	Citric acid (TCA) cycle intermediate; mitochondrial aconitase catalyzes conversion of cis‐aconitate to isocitrate; downregulation of aconitase activity with oxidative stress and aging impairs metabolism (Yan et al., 1997; Yarian et al., 2006) and decreases longevity (Cheng et al., 2015); aconitase‐null genotype related to reduced activity, shorter life, and neuronal cell death in Drosophila (Cheng et al., 2013)
Imidazole propionic acid	↓↓	↓↓	↓↓	↓↓	↓↓	↓↓	↓↓	Histidine metabolic product (microbial) and glutamate precursor; increased in diabetes and negatively impacts insulin signaling via reduction in IRS1 or IRS2 across multiple tissues (muscle, liver, and adipose) and mTORC1 signaling (Koh et al., 2018; Molinaro et al., 2020)
DMGV	↓	↑	↓↓	↓↓	↓↓	↓↓	↓↓	Implicated in hepatic steatosis, diabetes (O'Sullivan et al., 2017), and acute exercise responses (Nayor et al., 2020) and cardiovascular mortality (Ottosson et al., 2019)
Hydroxyproline	↓↓	↓	↓↓	↓↓	↓↓	↓↓	↓	Thought to reflect collagen metabolism; related to increased meat consumption (Pallister et al., 2016; Ross, Svelander, Undeland, Pinto, & Sandberg, 2015); increased cardiac hydroxyproline content with aging (Ito, Yamagiwa, & Sasaki, 1980)
Isocitrate	↓	↓↓	↓	↓↓	↓↓	↓	↓↓	Citric acid (TCA) cycle intermediate; deficiency of mitochondrial isocitrate dehydrogenase alters mitochondrial redox state (via NADPH), increases oxidative stress, and is thought to contribute to multi‐organ dysfunction (Han, Choi, Kim, Park, & Park, 2018; Kim et al., 2016; Kong, Han, Kim, Park, & Park, 2018; Ku, Ahn, Lee, Park, & Park, 2015; Lee, Kim, Park, Lee, & Park, 2016) and decreased lifespan (Cheng et al., 2015)
Leukotriene pathway metabolites (5‐HETE, 12‐HETE, 15‐HETE)	↓	↓	↓↓	↓↓	↓↓	↓↓	↓↓	HETEs (lipooxygenase products) linked to tissue inflammation (Kulkarni, Nadler, Mirmira, & Casimiro, 2021) and oxidative stress (Chen & Zou, 2022)
Glucuronic acid	↓	↓	↓↓	↓↓	↓↓	↓↓	↓↓	Involved in hepatic detoxification; associated with decreased longevity in mice and humans (Ho et al., 2019)
Cotinine	↓↓	↓↓	↓↓	↓↓	↓↓	↑	↓	Nicotine metabolite (smoking)

*Note*: Red colors represent positive associations and blue colors represent negative associations, with shades reflecting the strength of associations. “‐” represents absolute β < 0.003, “↑/↓” represents absolute beta between 0.003 and 0.01; “↑↑/↓↓” represents absolute β > 0.01.

^a^ Metabolite used for the heatmap.

### 3.3 Metabolome‐aging domain integration informs morbidity risk

**FIGURE 2:**
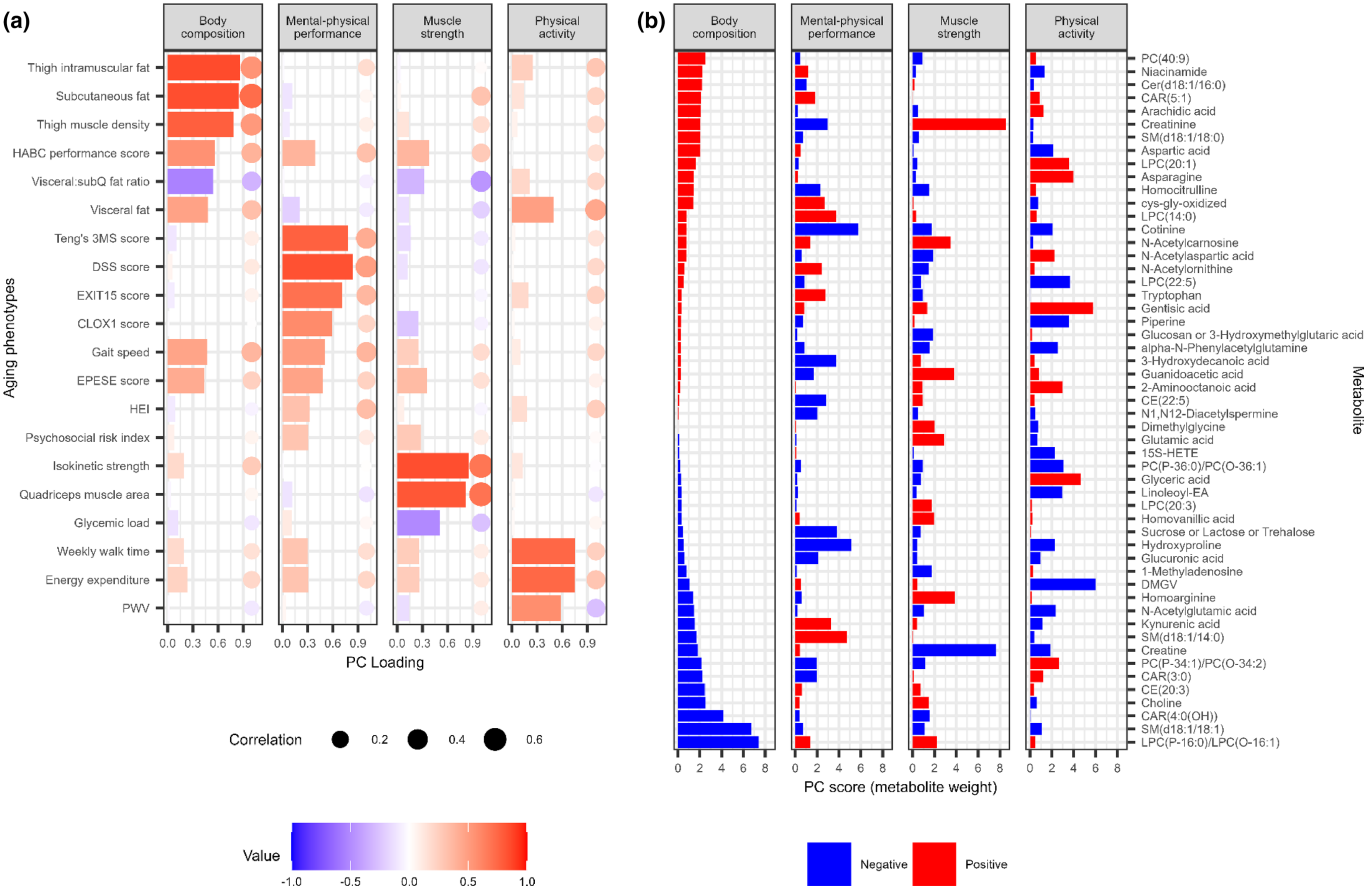
Unsupervised decomposition of metabolome‐aging phenome. (a) shows loadings [colorized bars] from a principal component analysis (PCA) of the relation between the metabolome and the aging phenome (as measured by LASSO regression), and the Pearson correlation [solid circles] between each generated metabolic score (for each aging domain by PCA) and the parent aging phenome. The absolute value of the loading on each phenotype is a measure of how closely the underlying metabolite is related to that phenotype. This approach decomposed the joint metabolite‐phenomic relations into four principal components (aging “axes”) encompassing 56% of the overall variation, corresponding to body composition (18%), mental‐physical performance (16%), muscle strength (12%), and physical activity (10%). The correlations between metabolic score and each parent phenotype are denoted as solid circles with size proportionate to the absolute value, which verify that the composite metabolite scores by the approach here were indeed related to the main phenotypes from which they were derived. (b) Selection of PC scores (weightings for metabolic scores) for the top 15 metabolites (by magnitude) included in each metabolic component and their weightings for other components. Relative weightings of each metabolite in each component were subsequently used to calculate a metabolite composite “score” as a quantitative index of that component. Red represents positive weighting and blue represents negative weighting.

Next, we used a combined LASSO regression‐PCA approach previously reported by our group (Murthy et al., 2020) to generate parsimonious “metabolic scores” of shared phenotypes of aging. The PCA approach across regression estimates between the metabolome (including 318 metabolites) and phenome (including 20 phenotypes) yielded four principal components (“axes” of aging) that described this joint metabolome‐phenome relation (≈56% total variance explained). The principal components were labeled based on the phenotypes with the highest absolute PC loading in each of the pre‐specified aging domains. For example, body composition measures such as thigh intermuscular fat, subcutaneous fat had the highest PC loadings in the first PC; Teng's 3MS score for cognitive function assessment and gait speed and EPESE score for physical performance measurement had the highest PC loadings in the second PC; Isokinetic strength and quadriceps muscle area that reflecting muscle strength and mass had the highest loadings for PC3; and physical activity measures were both with the highest loading for PC4. Figure 2a displays phenotype loadings for principal component (body composition, PC1; mental‐physical performance, PC2; muscle strength, PC3; physical activity, PC4) and correlations between metabolic scores and phenotypes. This approach also generated “weights” (PC scores) for metabolites that were combined for each PC to generate a composite metabolic score that reflected that PC (see Data S1 for metabolite weights [PC scores]; Figure 2b for top 15 metabolites for each component). As anticipated, the correlations between the metabolic scores and the parent phenotypes from which they were derived were strong and phenotypically consistent (e.g., body composition score correlated with CT metrics of adiposity; mental‐physical performance with mental scores and gait speed; muscle strength with muscle area and isokinetic strength, Figure 2a). In addition, the metabolic scores were not strongly related to age across the 10‐year age range in Health ABC (correlation coefficient ranged from −0.015 for body composition to 0.082 for physical activity). They also showed similar the sex and race differences to the parent phenotypes (Figure S3; Table S1). In general, White participants had higher measures and metabolic scores related to physical and cognitive function (e.g., Higher mental‐physical performance score, faster gait speed, higher Health ABC performance battery score, and higher Teng's 3MS score) than Black participants; Men had higher muscle strength and area (e.g., higher muscle strength score, higher Isokinetic strength, and higher quadriceps muscle area) than women.

**FIGURE 3:**
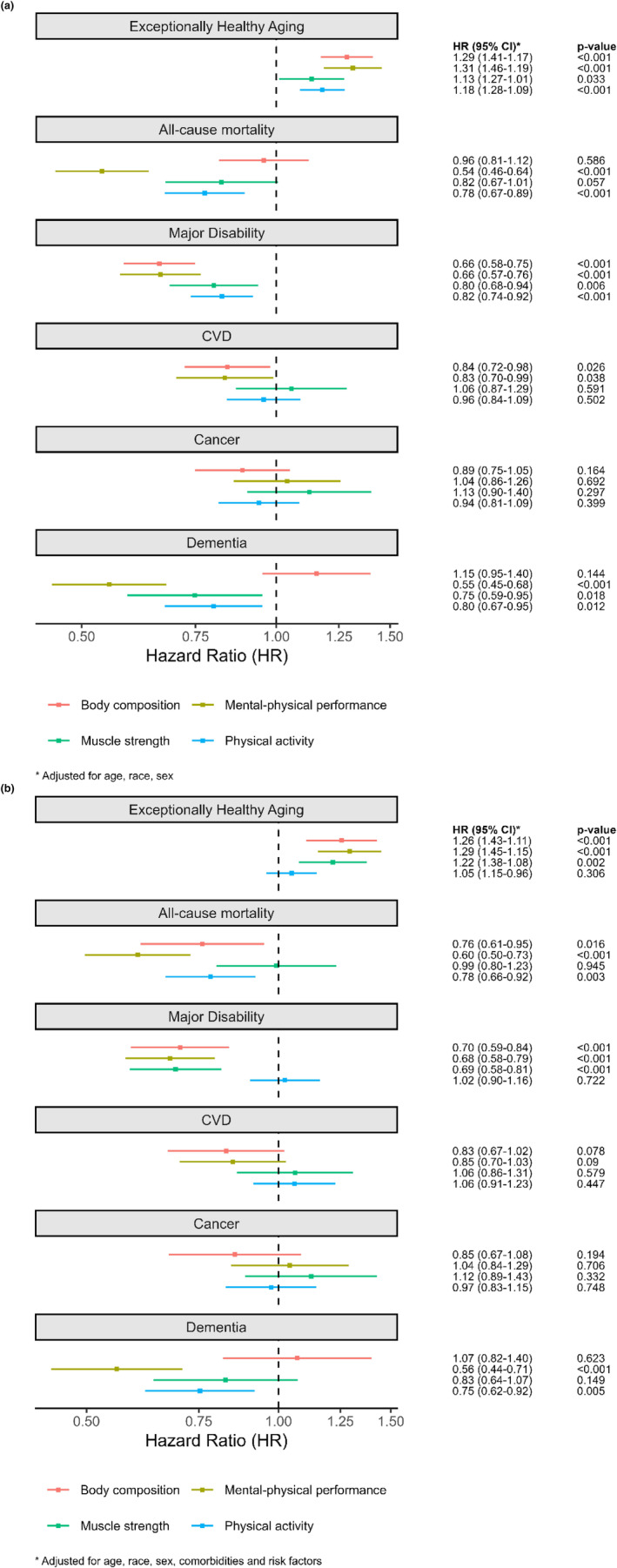
Metabolic scores (higher score indicates better health) and age‐related morbidity and mortality. Hazard ratios (HRs) are the relative hazard for outcome corresponding to one standardization higher metabolic scores. (a) Cox regression adjusted for age, sex, and race for mortality and a composite of exceptionally healthy aging (defined in text), as well as domain‐specific aging outcomes. (b) Results of Cox models fully adjusted for age, sex, race, comorbidities and risk factors. Full Cox model results are presented in Table S3.

We next studied the association of metabolic scores with cause‐specific morbidity and all‐cause mortality in 1246 Health ABC participants without CVD, cancer, dementia, and major disability prior to metabolite profiling. Overall, 515 individuals were “exceptionally healthy agers”, remaining free of age‐related morbidity development or death at a median 6.4 years follow‐up (25th–75th percentile 3.0–9.2 years). The overall cohort experienced a broad array of outcomes, including incident CVD (*n* = 250), cancer (*n* = 210), dementia (*n* = 163), major disability (*n* = 394), or death (*n* = 226), where non‐competing outcomes can co‐occur. We observed a generally consistent directionality of association (protective) between each of the 4 metabolic scores with “exceptionally healthy aging” (Figure 3a). That is, higher scores of mental‐physical performance (HR 1.29 [95% CI 1.15–1.45]), body composition (1.26 [1.11–1.43]), muscle strength (1.22 [1.08–1.38]), and physical activity (1.05 [0.96–1.15]) were associated with higher likelihood of experiencing exceptionally healthy aging although the association for physical activity was not statistically significant after full adjustment. When stratified by individual outcomes in this cohort, the metabolic scores exhibited a “protective” association with most clinical events, with results largely consistent across different components of exceptionally healthy aging (led by mental‐physical performance for all‐cause mortality HR 0.54 [95% CI 0.46–0.64]; full results in Table S3). Strikingly, associations were generally robust to full adjustment for comorbidities and commonly measured risk factors (Figure 3b).

### 3.4 Metabolic scores developed in older individuals display a wide range in a younger population and are strongly related to domain‐specific phenotypes and outcomes

**FIGURE 4:**
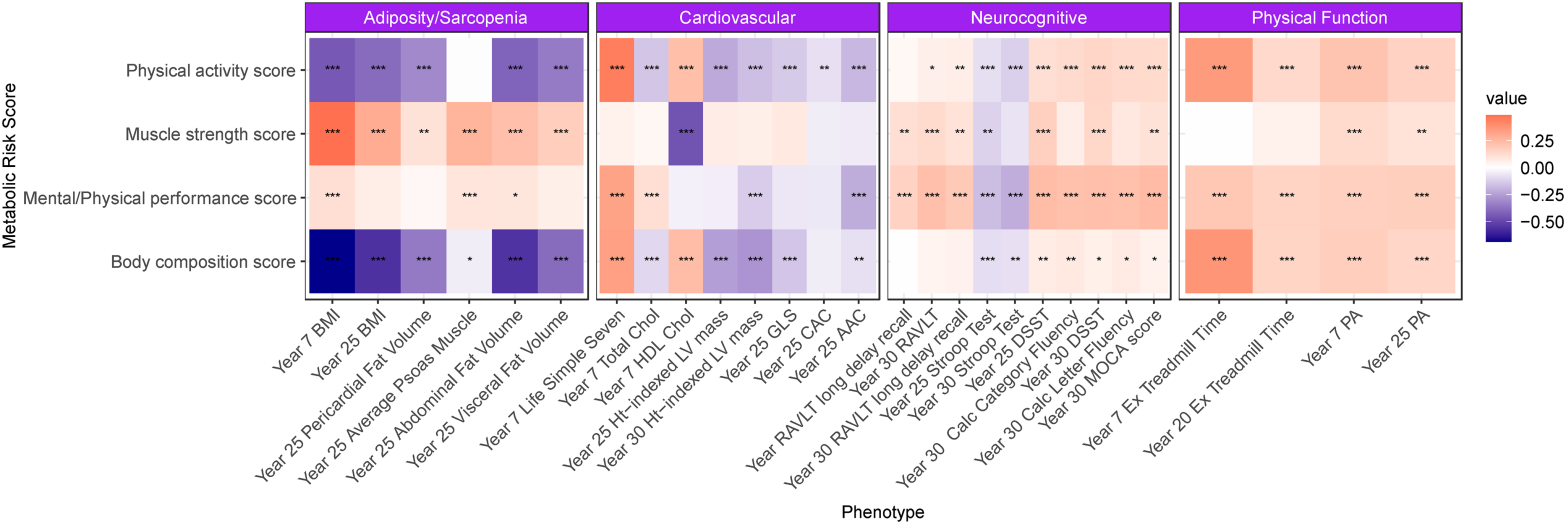
Metabolic risk scores in young adulthood are related to concurrent and future measures of age‐related phenotypes in CARDIA. The age‐, race‐, sex‐adjusted beta‐coefficients are displayed in the heatmap with positive value in coral and negative value in navy. *p < 0.05; **p < 0.01; ***p < 0.001. Full regression results available in Data S1.

A central question in our study was whether metabolic correlates of age‐related morbidity and phenotypic domains would exhibit broad variability in a population nearly four decades younger and whether that variability would be linked to subclinical risk of accelerated aging. Applying metabolite weights (PC scores) derived in Health ABC to CARDIA, we observed a similar race and sex distribution in CARDIA as observed in Health ABC four decades older (Figure S4). Importantly, each of the four scores showed variability in the CARDIA participants despite their younger age and less variation in age, suggesting metabolic contributions to aging domains are extant even at an earlier life stage. We subsequently found striking, domain‐specific relations of each of the four scores with phenotypes in CARDIA (Figure 4, full regression estimates in Data S1). Broadly, body composition and physical activity metabolic scores were related to measures of adiposity/sarcopenia, cardiovascular disease, and physical function—but not neurocognitive measures—in CARDIA, while the mental/physical performance score was related to neurocognitive measures and physical function, mirroring the associations in Health ABC.

**FIGURE 5:**
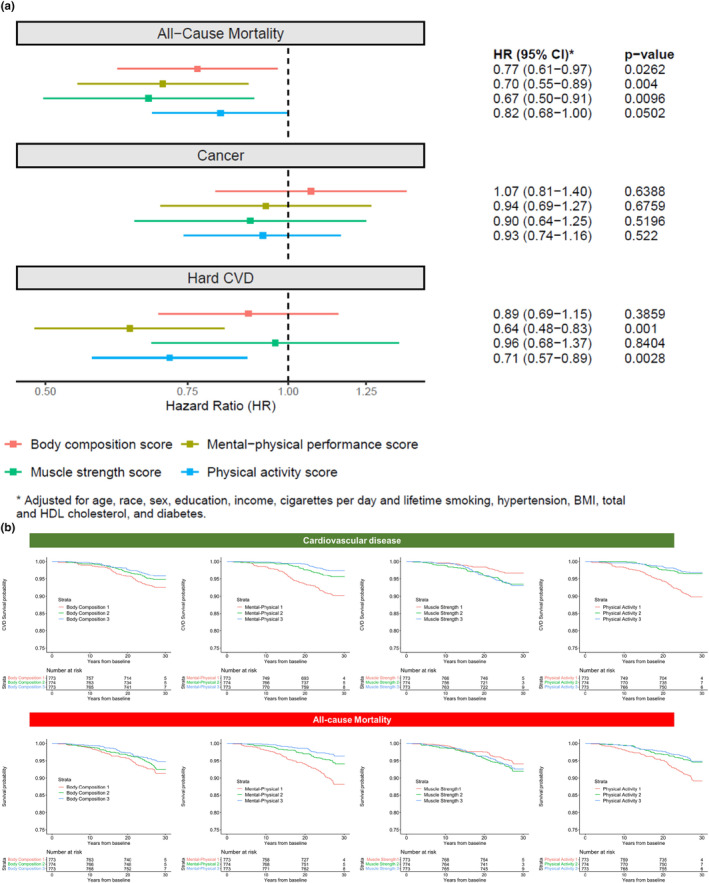
Metabolic risk scores in young adulthood are related to long‐term disease‐free survival over nearly three decades. (a) Shows the fully‐adjusted Cox estimates for three disease specific outcomes (cancer, CVD, and mortality). (b) Displays Kaplan–Meier survival estimates (by tertile of metabolic score) for CVD (left panel) and all‐cause mortality (right panel). Full Cox models are presented in Table S4.

At a median follow‐up of 27.2 years (25th–75th percentile 26.8–28.9 years) in CARDIA, we observed 155 mortality events, 113 cancer events, and 125 CVD events. The results of Cox regression are shown in Figure 5 (full regression in Table S4). In general, we observed strong associations between each metabolic score and mortality with better metabolic scores relating to lower mortality hazard after risk factors adjustment (ranged from muscle strength: HR 0.67 [95% CI 0.50–0.91] to body composition: 0.77 [0.61–0.97]). Physical activity score (0.71 [0.57–0.89]) and mental‐physical performance score (0.64 [0.48–0.83]) were associated with lower hazard of incident CVD. No statistically significant association between metabolic scores and incident cancer was observed.

## 4 DISCUSSION

Advances in treatment of acute lifespan‐limiting conditions (e.g., acute CVD, infectious disease, cancer) have directed efforts toward promoting healthy longevity by compressing morbidity to end of life to maximize quality of life. Here, we perform comprehensive metabolite profiling across the life‐course in two studies—the Health ABC and CARDIA studies—across multi‐dimensional aging‐related phenotypes in a broad age range, sex, and race. Integration of the metabolome and seven phenomic domains relevant to aging (including 20 aging‐related phenotypes) generated four major “axes” of aging (principal components) linked to the metabolome, reflecting body composition, mental‐physical performance, muscle strength, and physical activity. Metabolites prioritized by morbidity and organ function implicated a broad set of pathways relevant to energetics and nutrition, the host‐metagenomic relation, and inflammation and oxidative stress, many of which have not previously been described. Metabolic scores derived from this integrative approach were strongly and independently associated with cause‐specific morbidity over 9 years in Health ABC. In CARDIA—four decades younger—we replicated the association of these metabolic scores against subclinical multi‐organ morbidities and long‐term CVD and mortality over nearly three decades. Collectively, these results provide important evidence linking the metabolic state to multi‐organ morbidity development across the life‐course, providing a metabolic framework and resource to guide future mechanistic investigation and to develop biomarkers of age‐relevant metabolic dysfunction for future clinical study.

A key conceptual innovation in our approach was the integration of a 20‐dimensional phenotypic space with a broad metabolome to generate four complementary metabolic scores of aging, yielding several key observations. First, across the four generated metabolic scores, we found generally consistent associations of several scores—most clearly for the mental‐physical performance score—with composite “exceptionally healthy aging” and individual outcomes including mortality, disability, CVD, and dementia, but not cancer (Figure 3). Second, while several phenotypes were related to multiple other phenotypes (e.g., gait speed, cognitive measures), the metabolome‐phenome integration generally prioritized phenotype‐score combinations well described in the literature (Figure 2): for example, mental‐physical score encompassed measures of cognitive function and gait speed, consistent with emerging evidence linking neurocognitive impairment, dementia, and functional decline (Ikegami et al., 2019; Tian et al., 2017). Third, we were surprised to see similar associations in a four decades younger cohort, the CARDIA study, as it is known that the associations between metabolites and age‐related morbidities are malleable. In both cohorts, specific metabolic scores were consistently associated with their cognate subclinical phenotypes (e.g., mental‐physical metabolic scores to neurocognitive function) but not other phenotypes of interest (e.g., body composition metrics). Collectively, these results suggest that a metabolic snapshot may capture susceptibility to multi‐morbidity across age (and potentially even independent of it), supporting a generalizable application of metabolic function assessments in prioritizing mechanistic targets for study or intervenable biomarkers of therapeutic response in aging.

The broad metabolic quantification in this study also afforded us the opportunity to interrogate potential mechanisms of age‐related morbidity, specifically around cardiometabolic health. Prior data in this field has focused on age‐dependence of the metabolome (Chaleckis et al., 2016; Dunn et al., 2015; Menni et al., 2013; Swann et al., 2013; Yu et al., 2012) or select cross‐sectional aging phenotypes (Swann et al., 2013). The multi‐dimensional phenotyping in Health ABC allowed us to investigate shared and divergent metabolic pathways across phenotypes. Our findings were consistent with studies at the extremes of age, which have implicated broad metabolic mechanisms of oxidative stress and inflammation (e.g., via bioactive lipids, arachidonic acid metabolites, and tryptophan) in the oldest old (Collino et al., 2013; Montoliu et al., 2014). In addition, the heterogeneity in metabolic scores and their relation to outcome in a synchronized age cohort (Health ABC) addresses the large amount of unexplained variation in studies attempting to “predict” chronologic age with the metabolome (*R* ^2^ for age 60%–70%; Rist et al., 2017), effectively underscoring that metabolic heterogeneity tied to morbidity may be important beyond chronologic age itself. This extends prior evidence targeting biological age (e.g. “immune age”, “calcium‐adjusted age”) as a higher precision measure than chronological age to metabolism (Alpert et al., 2019; Shaw et al., 2006). Indeed, efforts to determine biological age via association with age‐related morbidity uncover similar findings as in our study, including metabolites indicative of mitochondrial beta‐oxidation, citric acid cycle flux, and several microbial byproducts (e.g., indole) (Johnson et al., 2019). Furthermore, the replication in CARDIA cohort four decades younger suggests that despite twin studies supporting an important influence of environment on age‐related metabolic change (Bunning et al., 2020), select similar metabolic states may influence rates of aging across the life‐course at least as early as the third decade of life, independent of traditional risk factors at baseline. Nevertheless, it is also possible that unmeasured confounding factors contributed to similar associations between metabolism and aging‐related outcomes across the two cohorts. Further validation across diverse cohorts will be necessary to assess generalizability of our findings and their provocative implications.

This work is an important advance in the relation of human metabolism to healthy aging. The longitudinal design of both Health ABC and CARDIA—alongside the richness of multi‐parametric phenotyping on age‐related potential for reverse causation in the relation of morbidities beyond current knowledge—limits the metabolism to compression of morbidity. The 20 phenotypes included in our study encompass broad morphometric, functional, and environmental mechanisms of aging—from tissue distributions and muscle strength to dietary patterns, energy expenditure, and activity. While longitudinal assessment of the metabolome with age or perturbation (e.g., training, exercise) may further prioritize the relation between metabolic pathways and select phenotypes (e.g., muscle strength) (Ahadi et al., 2020), validation within CARDIA suggests there could be a consistent link between certain metabolic pathways and disease across age. These results also suggest that survival biases inherent in studying older adults may not bias our result. It is notable that cotinine (a metabolic hallmark of cigarette smoking exposure) was strongly weighted in the mental‐physical performance score, which strongly validated in CARDIA against outcomes and phenotypes. While cotinine may provide a precise estimate of short‐term smoking exposure, our outcome models in both cohorts accounted for these exposures using standard self‐reported measures. In addition, most studies of metabolic aging include individuals of European descent (Panyard et al., 2022), while samples in Health ABC included ~40% Black participants and both sexes, offering a broader ability to address race‐ and sex‐specific variability of morbidity‐related metabolic patterns. In addition, this study was amongst the largest studies to date utilizing LC‐MS techniques to quantify circulating metabolome, providing an invaluable resource to the scientific community for prioritizing targets for downstream discovery (including eventually studies in metabolites not currently annotated). Nevertheless, this study has limitations. In spite of moderate effect sizes for certain individual metabolites, we did not identify significant metabolite associations with psychosocial stress after accounting for multiple comparisons, which might be due to lack of power. Future studies employing targeted‐approaches for the metabolic mechanisms underlying psychosocial stress may be helpful in this regard. The LC‐MS techniques provide relative quantification of the metabolome across participants. Extension to absolute measures of metabolite levels would be of interest for future efforts.

In conclusion, in a large group of White and Black older Americans, we identified metabolic patterns of multiple, diverse aging phenotypes which specify conserved and novel pathways of metabolism relevant to systems‐wide aging and long‐term health outcomes in both older and younger adults. Metabolic signatures derived from the healthiest of the older population were consistently related to lower subclinical multi‐organ morbidities and long‐term CVD and mortality risk across age in younger individuals, implicating metabolic processes in healthy systems‐wide aging across the life course. Metabolic biomarkers of human aging may represent relevant targets of prognosis and diagnosis across the lifespan, potentially informing responses to therapeutic/lifestyle intervention.

## AUTHORS CONTRIBUTIONS

Shanshan Yao analyzed data, constructed illustrations, and wrote the manuscript. Laura A. Colangelo analyzed data, reviewed, and revised manuscript. Andrew S. Perry, Megan M. Marron, Kristine Yaffe, Sanaz Sedaghat, Joao A.C. Lima, and Qu Tian discussed, reviewed, and revised the manuscript. Clary B. Clish performed the assay. Anne B. Newman, Ravi V. Shah, and Venkatesh L. Murthy conceptualized the study, reviewed, and revised the manuscript.

## FUNDING INFORMATION

This work was supported by National Institute on Aging Contracts N01‐AG‐6–2101, N01‐AG‐6–2103, and N01‐AG‐6–2106; National Institute on Aging Grant R01‐AG028050; National Institute of Nursing Research Grant R01‐NR012459; National Heart, Lung, and Blood Institute Grant R01‐HL136685. The Coronary Artery Risk Development in Young Adults Study (CARDIA) is conducted and supported by the National Heart, Lung, and Blood Institute (NHLBI) in collaboration with the University of Alabama at Birmingham (HHSN268201800005I & HHSN268201800007I), Northwestern University (HHSN268201800003I), University of Minnesota (HHSN268201800006I), and Kaiser Foundation Research Institute (HHSN268201800004I). The project was also supported by was supported by grant R01‐HL098445 from the National Heart, Lung, and Blood Institute (NHLBI) to Vanderbilt University and Wake Forest University. The CARDIA cognitive function ancillary study is supported by NIA R01AG063887. This work was also supported in part by the Intramural Research Program of the National Institute on Aging. Metabolomics were funded by National Institute on Aging (R01‐AG‐059729). MMM is supported by the National Institute on Aging K01‐AG‐075143. VLM is funded by the Melvyn Rubenfire Professorship in Preventive Cardiology. ABN is supported by the Pittsburgh Pepper Center P30 AG024827 and the UPMC Endowed chair in Geroscience.

## CONFLICT OF INTEREST STATEMENT

Dr. Murthy owns stock in General Electric. He has received research funding through his institution as well as personal consulting fees from Siemens. He is also a paid scientific advisor for INVIA Medical Imaging Solutions.

## Supporting information


Appendix S1.

## Data Availability

Data supporting this study are available from https://healthabc.nia.nih.gov/.
