# Supplementary material for: Implications of metabolism on multi‐systems healthy aging across the lifespan
Source: Aging Cell. 2024 Jan 29;23(4):e14090. doi: 10.1111/acel.14090 (PMC11019145; doi:10.1111/acel.14090)
Supplement: Supplementary file 1 — Appendix S1. [file ACEL-23-e14090-s001.docx]

**Supplement for Shanshan et al. “**Implications of metabolism on multi-systems aging across the lifespan**”**

1. **Supplementary Methods**
2. **Supplementary Tables**
3. **Supplementary Figures**
4. **Excel file of regression estimates (“Online Only”)**

**Supplementary Methods**

**Two-phase LASSO regression-PCA approach**

Similar to our previous work^1^, we joined two powerful methods to derive metabolite signatures across multiple phenotypic measures: (1) the construction of parsimonious models for each phenotype as a function of metabolites using **Least Absolute Shrinkage and Selection Operator (LASSO) regression** to minimize over-fitting and (2) aggregation of the results of these regressions with **principal components analysis (PCA)**. The overall workflow of a LASSO-PCA approach and the detailed methodologies are described below.

**Overall workflow (Steps 2-5 in Supplementary Figure 1)**

***Step 2:*** *Variable pruning* for Health ABC study and CARDIA overlapped metabolites (n=478) based on absolute Pearson correlation until the maximum Pearson correlation between all remained metabolites <=0.8.

***Step 3:*** *LASSO regressions* were performed for each of the 20 phenotypes as dependent variable with all metabolite remained after variable pruning (n=318) as predictors.

***Step 4:*** *PCA* performed on the shrunken coefficients in matrix of phenotypes-metabolites associations with phenotypes in the columns and metabolites in the rows and penalized beta-coefficients in the cells. All metabolites with at least one beta coefficient not equal to 0 across 20 phenotypes in the LASSOs were included (n=318). Based on a scree plot of the variance explained by each PC, the number of PCs were specified (n=4) and each of the selected PCs is labeled according to the loadings of each aging phenotype on that PC.

***Step 5:*** PC score for each of the 318 metabolites for each of the selected 4 PCs were derived as the metabolite weightings. The PC scores (metabolite weights) and the value of metabolites are used to calculate metabolic scores (n=4) for each participant, which are calculated as the sum of metabolite levels weighted by PC score (metabolite weights) of metabolites.

**LASSO regression**

Traditional least squares regression has been considered inefficient for high dimensional data (e.g. metabolomic data) as it has difficulty with handling collinear metabolites and overfitting. In addition, traditional least squares linear models provide a 𝛽 coefficient for every metabolite included in a model, whether or not that predictor explains meaningful variance in the phenotype. This absence of parsimony can contribute to complex multivariable models that are difficult to apply in other datasets due to overfitting and lack of complete data^1^.

Penalized (also referred to as regularized) regression methods (LASSO, ridge, elastic net) have been developed to reduce overfitting and, in the case of LASSO and elastic net, provide parsimonious model. In this study, we used LASSO regression, which adds a penalty term for the number of predictors as well as shrinks to estimated coefficients toward zero. Although LASSO reduces overfitting that may occur in linear regression but does not work the best with multi-collinearity. Therefore, in this study, we did variable pruning prior to the LASSO regression based on the correlations between metabolites. The absolute values of pair-wise correlations are considered. If two variables have a high correlation, the variable with the largest mean absolute correlation will be removed. A Pearson correlation of >0.8 was considered highly correlated. The severity of penalization is controlled by a hyperparameter lambda (𝜆). Selection of optimal values for the hyperparameters 𝜆 is accomplished by 10-fold cross-validation repeated 10 times. That is, we repeatedly (10 times) randomly split the Health ABC cohort into 10 folds and used each of the 10 sets as testing set and the other 9 sets combined as training set to minimize overfitting (10-fold cross-validation) and optimize LASSO model fit.

**Principal components analysis (PCA)**

In this approach, we generated a metabolome-phenome matrix with each phenotype in a column, each metabolite selected by the LASSO regressions in rows, and the cells filled with the shrunk beta coefficients. We have presented this matrix as a heatmap in the main text. As mentioned in the main text, the principal scores for each metabolite according to their weight on each phenotype-based principal component were used as the weight for metabolite composite scores. The metabolite composite scores were summation of the “weights” multiplied with standardized metabolite concentration. For survival analysis, standardized metabolites scores were used.

**Sensitivity analysis**

There were two batches of metabolites measurement due to lock down during the COVID-19 pandemic and there are only very slight batch differences. Therefore, we also did a sensitivity analysis after accounting for the batch effect. First, single metabolite regression was repeated for all the participants with different models: (1) ordinary linear regression model without adjusting for different batches; (2) linear regression with adjustment of batches; (3) linear regression with adjustment of batch and metabolite-batch interaction. Besides, we also run ordinary linear regression stratified by batch and synthesize the beta coefficients with a meta-regression model. Beta-coefficients from the latter three models were highly close to and correlated with the ones from the first model. Second, we also imputed the missing metabolite data with half of the minimum value in each batch. Following that, we replicated the above models and find almost similar beta-coefficients, principal components, and hazard ratios for survival analysis. Therefore, to simplify the analysis, we finally analyze the data without considering the batch effect.

**Phenotype measurements**

These sections were directly taken with minimal modification from previous references for fidelity of scientific reproduction and increased rigor and reproducibility. The references here provide attribution to this reproduction^2-11^.

- **Physical function domain**: Physical function included gait speed at Year 2 visit, the Epidemiologic Studies of the Elderly (EPESE) battery, and the Health ABC performance battery at baseline. Gait speed was assessed over a 20-meter straight course set up in a corridor and timed from the first footfall over the starting line to the first footfall over the finish line^11^. The EPESE battery consists of five repeated timed chair stands, timed standing balance (with feet in parallel, semi-tandem, and tandem positions), and a 6-m walk to determine usual gait speed^9,10^. The Health ABC performance battery was expanded EPESE battery with the hold times on the standing balance items being increased to 30 seconds, and an additional standing balance position, single-leg stance, and a narrow walk test added^9,10^.
- **Neurocognitive/Executive Function domain**: Neurocognitive function was measured using the Teng-modified Mini-Mental Status Exam (3MS)^8^ and the digit symbol substitution test (DSST)^8^ at baseline. Executive control function was measured by a scored clock drawing task (CLOX 1) and the 15-item Executive Interview (EXIT 15), a shortened version of the 25-item Executive Interview developed for the Health ABC study, both at Year 3 visit^11^.
- **Adiposity/Sarcopenia domain**: Body composition phenotypes measured at baseline including thigh intermuscular fat area, quadriceps muscle area, visceral adipose tissue, subcutaneous adipose tissue, and thigh muscle density were measured by computed tomography scans^4-7^. Visceral-to-subcutaneous adiposity ratio was calculated as visceral fat area divided by subcutaneous fat area. Isokinetic strength of the knee extensors was determined at 60°/s using the Kin-Com dynamometer at Year 2 visit^10^.
- **Vascular aging domain**: Baseline arterial pulse wave velocity (A-PWV) was measured using simultaneous Doppler flow signals obtained from the right carotid and right femoral arteries using nondirectional transcutaneous Doppler flow probes (Park Medical Electronics Inc; Model 810A, 9.0–10.0-MHz probes)^5^ to calculate the velocity.
- **Dietary pattern domain**: A Healthy Eating Index (HEI) score and daily glycemic load summary variables were calculated based on the modified Block Food Frequency Questionnaire (FFQ) that was administered at the Year 2 follow-up visit^3^.
- **Psychosocial stress domain**: Psychosocial risk index consisting of depressive symptoms, anxiety symptoms, negative life events, and inadequate emotional support was constructed based on data collected at baseline visit^2^. The scores for four domains were: Depression (score 0-60; CES-D depression scale); (2) social anxiety (0-9; based on questions “During the past week, have you felt nervous or shaky inside? Fearful? Tense or keyed up?”); (3) negative life events (0-7; based on accident/death in close friend, family, pets, or changes in relationships, assault/physical violence, legal difficulty); (4) inadequate social network (0-1; based on needs for additional emotional support). Measures are standardized to 0-1 (CES-D/60; social anxiety/9; negative life events/7) and summed as an index of psychosocial stress (0-4).
- **Physical activity domain**: Physical activity measurements included total minutes walked per week and a calculated energy expenditure (kcal/kg/week) in walking and climbing stairs at Year 2 visit. To calculate energy expenditure in climbing stairs, we assigned 4.0 kcal/kg/hour of stair climbing plus an additional 1.0 kcal/kg/hour carrying a load like laundry, groceries, or an infant. For energy expenditure in walking for exercise, we assigned 4.0 kcal/kg/hour walking briskly, 3.0 to walking at moderate pace, and 2.0 to strolling.

**Incident events**

These sections were directly taken with minimal modification from previous references for fidelity of scientific reproduction and increased rigor and reproducibility. The references here provide attribution to this reproduction^12,13^.

- **Exceptionally healthy aging** was defined as surviving to Year 10 visit without developing CVD, cancer of any type, dementia, or major disability. Time to event was started from the year 2 examination. Information about incident events, including reported new diagnoses and hospitalizations, were collected by in-person examination alternating with a telephone interview every 6 months. Cause of death was adjudicated by a panel of clinicians who reviewed all hospital medical records, death certificates, informant interviews, and autopsy reports^13^. Date of death was taken from the death certificate or national death index. Overnight hospitalizations were reviewed at each site by local adjudicators for adjudication of non-fatal cardiovascular disease (CVD), cancer, and fracture^13^.
- **Incident CVD** was defined as incident myocardial infarction, angina pectoris, congestive heart failure and/or stroke resulting in symptoms and/or treatment based on data of an overnight hospitalization, or coronary and/or stroke death during follow up.
- **Incident cancer** included any cancer, except non-melanoma skin cancer, validated with a pathology report.
- **Incident dementia** was adjudicated by an expert committee composed of a same panel of experienced medical doctors during the whole follow-up period^12^. This committee identified dementia diagnosis on medical records using a predefined adjudication form. Dementia was defined by any of the following criteria: (i) ≥ 1.5 standard deviation (SD) race-stratified 3MS decline from baseline visit to last available examination; (ii) dementia as primary or secondary diagnosis of admission on hospital records with 3MS ≤ 90 points (participants were asked about hospital admissions twice a year); (iii) prescription of dementia drug (donepezil, galantamine, memantine, rivastigmine or tacrine) recorded on the yearly drug inventory. Date of first available record of dementia diagnosis was used to define onset. Major disability event was defined as incident needing equipment, having mobility disability or needing help with an activity of daily living.

**Covariates**

We used self-reported age, race, highest education level, and smoking status at baseline. Height and weight were recorded at the Year 2 visit. Baseline history or presence of hypertension and diabetes were based on self-report of a physician diagnosis or taking medication for the disease. Participants brought all prescription medications used in the last two weeks to Year 2 visit for medication inventory. Cystatin C was measured in serum at baseline by the Laboratory for Clinical Biochemistry Research at the University of Vermont.

**REFERENCES**

1. Murthy VL, Reis JP, Pico AR, et al. Comprehensive Metabolic Phenotyping Refines Cardiovascular Risk in Young Adults. *Circulation* 2020; **142**(22): 2110-27.

2. Vogelzangs N, Beekman AT, Kritchevsky SB, et al. Psychosocial risk factors and the metabolic syndrome in elderly persons: findings from the Health, Aging and Body Composition study. *J Gerontol A Biol Sci Med Sci* 2007; **62**(5): 563-9.

3. Lee JS, Kritchevsky SB, Tylavsky FA, et al. Weight-loss intention in the well-functioning, community-dwelling elderly: associations with diet quality, physical activity, and weight change. *Am J Clin Nutr* 2004; **80**(2): 466-74.

4. Beavers KM, Beavers DP, Houston DK, et al. Associations between body composition and gait-speed decline: results from the Health, Aging, and Body Composition study. *Am J Clin Nutr* 2013; **97**(3): 552-60.

5. Abbatecola AM, Chiodini P, Gallo C, et al. Pulse wave velocity is associated with muscle mass decline: Health ABC study. *Age (Dordr)* 2012; **34**(2): 469-78.

6. Huynh K, Ayers C, Butler J, et al. Association Between Thigh Muscle Fat Infiltration and Incident Heart Failure: The Health ABC Study. *JACC Heart Fail* 2022; **10**(7): 485-93.

7. Madero M, Katz R, Murphy R, et al. Comparison between Different Measures of Body Fat with Kidney Function Decline and Incident CKD. *Clin J Am Soc Nephrol* 2017; **12**(6): 893-903.

8. Rosano C, Simonsick EM, Harris TB, et al. Association between physical and cognitive function in healthy elderly: the health, aging and body composition study. *Neuroepidemiology* 2005; **24**(1-2): 8-14.

9. Simonsick EM, Newman AB, Nevitt MC, et al. Measuring higher level physical function in well-functioning older adults: expanding familiar approaches in the Health ABC study. *J Gerontol A Biol Sci Med Sci* 2001; **56**(10): M644-9.

10. Brach JS, Simonsick EM, Kritchevsky S, et al. The association between physical function and lifestyle activity and exercise in the health, aging and body composition study. *J Am Geriatr Soc* 2004; **52**(4): 502-9.

11. Atkinson HH, Rosano C, Simonsick EM, et al. Cognitive function, gait speed decline, and comorbidities: the health, aging and body composition study. *J Gerontol A Biol Sci Med Sci* 2007; **62**(8): 844-50.

12. Aubert CE, Bauer DC, da Costa BR, et al. The association between subclinical thyroid dysfunction and dementia: The Health, Aging and Body Composition (Health ABC) Study. *Clin Endocrinol (Oxf)* 2017; **87**(5): 617-26.

13. Kalogeropoulos AP, Georgiopoulou VV, Murphy RA, et al. Dietary sodium content, mortality, and risk for cardiovascular events in older adults: the Health, Aging, and Body Composition (Health ABC) Study. *JAMA Intern Med* 2015; **175**(3): 410-9.

**Supplementary Tables**

Supplementary Table 1. Phenotypes of participants from the Health ABC study. Abbreviations: n, number where these covariates were available; SD, standard deviation. The abbreviations for the phenotypes are shown in Table 1. All the phenotypes statistically significantly differ across the four race/sex groups using one-way ANOVA tests (p<0.05).

| **Phenotypes** | **n** | **Overall** | **Black men** | **Black women** | **White men** | **White women** |
| --- | --- | --- | --- | --- | --- | --- |
|  |  | **Mean (SD)** | | | | |
| 20-meter gait speed (m/sec) | 2,412 | 1.1 (0.2) | 1.1 (0.2) | 1.0 (0.2) | 1.2 (0.2) | 1.2 (0.2) |
| EPESE (0-12) | 2,432 | 10.1 (1.5) | 10.0 (1.4) | 9.5 (1.7) | 10.6 (1.3) | 10.1 (1.4) |
| Health ABC performance battery (0-4) | 2,377 | 2.2 (0.5) | 2.2 (0.5) | 1.9 (0.5) | 2.5 (0.5) | 2.2 (0.5) |
| Teng 3MS (0-100) | 2,462 | 90.6 (7.9) | 85.0 (9.8) | 87.6 (8.7) | 92.4 (6.0) | 93.8 (5.3) |
| EXIT15 (0-30) | 2,206 | 6.6 (4.2) | 9.0 (4.6) | 7.8 (4.8) | 5.9 (3.6) | 5.4 (3.4) |
| CLOX1 (0-15) | 2,198 | 10.7 (2.4) | 9.8 (2.6) | 10.4 (2.3) | 10.7 (2.6) | 11.3 (2.0) |
| DSS | 2,440 | 36.3 (14.4) | 24.8 (13.8) | 30.5 (14.6) | 39.4 (11.7) | 43.5 (11.5) |
| Total quadriceps muscle area (cm^2^) | 2,418 | 103.5 (26.5) | 128.7 (22.9) | 91.9 (16.4) | 119.6 (18.4) | 78.9 (12.9) |
| Isokinetic strength (N-m) | 2,117 | 104.7 (37.9) | 131.0 (37.1) | 83.0 (24.7) | 128.8 (31.3) | 76.5 (20.1) |
| Average thigh muscle density (Hounsfield units) | 2,418 | 35.6 (6.9) | 37.2 (6.4) | 32.5 (7.1) | 37.4 (6.3) | 34.9 (6.7) |
| thigh intermuscular fat area (cm^2^) | 2,418 | 20.3 (12.8) | 22.0 (16.1) | 25.8 (14.3) | 18.8 (11.7) | 17.3 (8.8) |
| Abdomen subcutaneous fat area (cm^2^) | 2,310 | 282.0 (120.0) | 230.3 (98.1) | 379.0 (135.1) | 225.2 (80.9) | 308.1 (104.2) |
| Abdomen visceral fat area (cm^2^) | 2,377 | 143.2 (67.2) | 127.0 (64.6) | 130.0 (57.9) | 169.4 (71.0) | 131.9 (61.4) |
| Abdomen Visceral:SubQ fat ratio | 2,310 | 0.6 (0.3) | 0.6 (0.3) | 0.4 (0.2) | 0.8 (0.3) | 0.4 (0.2) |
| PWV (cm/sec) | 2,008 | 901.1 (384.4) | 946.2 (392.7) | 927.2 (433.4) | 911.1 (365.2) | 849.0 (360.1) |
| HEI score (0-100) | 2,416 | 69.6 (12.1) | 63.2 (12.2) | 68.8 (11.9) | 70.6 (11.3) | 72.4 (11.8) |
| Daily glycemic load | 2,427 | 130.4 (56.2) | 151.8 (72.6) | 126.8 (55.9) | 138.7 (54.0) | 111.5 (39.8) |
| Psychosocial Risk Index (0-4) | 2,359 | 0.5 (0.5) | 0.5 (0.5) | 0.5 (0.5) | 0.4 (0.4) | 0.5 (0.5) |
| Weekly walk time (mins) | 2,465 | 135.1 (261.3) | 132.6 (265.9) | 84.3 (179.2) | 170.6 (301.7) | 132.8 (253.3) |
| Energy expenditure (kcal/kg/week) | 2,467 | 7.5 (14.0) | 7.1 (13.2) | 4.6 (9.2) | 9.6 (16.4) | 7.6 (14.0) |

**Supplementary Table 2**. Characteristics of Aging phenotypes in CARDIA. Reported as mean (standard deviation). Year in CARDIA of measurement is noted. Abbreviations for phenotypes in **Table 1**.

| Characteristic | N | Mean (SD) |
| --- | --- | --- |
| Year 20 Exercise Treadmill Time (min) | 1,629 | 7.8 (2.7) |
| Year 25 Digit Symbol Substitution Test | 1,828 | 71 (16) |
| Year 25 RAVLT long delay recall | 1,822 | 8.4 (3.2) |
| Year 25 Stroop Test (interference score) | 1,829 | 22 (10) |
| Year 30 RAVLT long delay recall | 1,705 | 8.6 (3.4) |
| Year 30 Stroop Test (interference score) | 1,666 | 22 (11) |
| Year 30 RAVLT | 1,701 | 9.2 (1.9) |
| Year 30 Calculated Category Fluency | 1,691 | 21 (6) |
| Year 30 Calculated Letter Fluency | 1,671 | 42 (13) |
| Year 30 Digit Symbol Substitution Test | 1,692 | 69 (17) |
| Year 30 MOCA score | 1,693 | 24 (4) |
| Year 25 Pericardial Fat Volume (ml) | 1,717 | 57 (34) |
| Year 25 Average Psoas Muscle Volume (cm^3^) | 1,714 | 12 (4) |
| Year 25 Abdominal Fat Volume (cm^3^/10 mm) | 1,715 | 454 (204) |
| Year 25 Visceral Fat Volume (cm^3^/10 mm) | 1,715 | 129 (72) |
| Year 25 Height-indexed left ventricular mass (g/m^2.7^) | 1,762 | 39 (11) |
| Year 30 Height-indexed left ventricular mass (g/m^2.7^) | 1,504 | 40 (11) |
| Year 7 Exercise Treadmill Time (min) | 2,118 | 9.7 (2.7) |
| Year 7 CARDIA Physical Activity Score (Activity units) | 2,299 | 375 (282) |
| Year 25 CARDIA Physical Activity Score (Activity units) | 1,878 | 372 (284) |
| Year 25 Coronary artery calcium score (Agatston score) | 1,723 | 35 (144) |
| Year 25 Abdominal aortic calcium score (Agatston score) | 1,687 | 156 (475) |
| Year 25 Global longitudinal strain | 1,677 | -15.2 (2.4) |
| Year 7 Body mass index (kg/m^2^) | 2,320 | 25.7 (5.0) |
| Year 25 Body mass index (kg/m^2^) | 1,889 | 29.2 (6.4) |
| Year 7 HDL Cholesterol (mg/dL) | 2,320 | 54 (14) |
| Year 7 Total Cholesterol (mg/dL) | 2,320 | 176 (33) |
| Year 7 Life Simple Seven Score | 2,205 | 10.2 (1.8) |

**Supplementary Table 3. Cox regression results for Exceptionally Healthy Aging and its individual components in Health ABC.** Minimally adjusted model was with adjustment for age, sex, and race; Fully adjusted model was with further adjustment for comorbidities and commonly measured risk factors. Statistically significant results were denoted in bold. Hazard ratio and 95% confidence interval (HR [95%CI]) expressed per standard deviation of the score.

| **Outcome** | **Metabolic score** | **Unadjusted** | | **Minimally adjusted** | | **Fully adjusted** | |
| --- | --- | --- | --- | --- | --- | --- | --- |
|  |  | **HR [95%CI]** | **P value** | **HR [95%CI]** | **P value** | **HR [95%CI]** | **P value** |
| CVD | Body composition | 0.99 [0.88, 1.12] | 0.907 | **0.84 [0.72, 0.98]** | **0.026** | 0.83 [0.67, 1.02] | 0.078 |
|  | Mental-physical performance | 0.90 [0.79, 1.01] | 0.081 | **0.83 [0.70, 0.99]** | **0.038** | 0.85 [0.70, 1.03] | 0.090 |
|  | Muscle strength | **1.18 [1.05, 1.33]** | **0.008** | 1.06 [0.87, 1.29] | 0.591 | 1.06 [0.86, 1.31] | 0.579 |
|  | Physical activity | 0.96 [0.84, 1.09] | 0.525 | 0.96 [0.84, 1.09] | 0.502 | 1.06 [0.91, 1.23] | 0.447 |
| Cancer | Body composition | 1.08 [0.94, 1.24] | 0.257 | 0.89 [0.75, 1.05] | 0.164 | 0.85 [0.67, 1.08] | 0.194 |
|  | Mental-physical performance | 0.98 [0.86, 1.13] | 0.783 | 1.04 [0.86, 1.26] | 0.692 | 1.04 [0.84, 1.29] | 0.706 |
|  | Muscle strength | **1.35 [1.17, 1.54]** | **<0.001** | 1.13 [0.90, 1.40] | 0.297 | 1.12 [0.89, 1.43] | 0.332 |
|  | Physical activity | 0.93 [0.80, 1.07] | 0.285 | 0.94 [0.81, 1.09] | 0.399 | 0.97 [0.83, 1.15] | 0.748 |
| Dementia | Body composition | 0.96 [0.82, 1.12] | 0.586 | 1.15 [0.95, 1.40] | 0.144 | 1.07 [0.82, 1.40] | 0.623 |
|  | Mental-physical performance | **0.65 [0.56, 0.74]** | **<0.001** | **0.55 [0.45, 0.68]** | **<0.001** | **0.56 [0.44, 0.71]** | **<0.001** |
|  | Muscle strength | **0.83 [0.71, 0.96]** | **0.015** | **0.75 [0.59, 0.95]** | **0.018** | 0.83 [0.64, 1.07] | 0.149 |
|  | Physical activity | **0.83 [0.70, 0.98]** | **0.027** | **0.80 [0.67, 0.95]** | **0.012** | **0.75 [0.62, 0.92]** | **0.005** |
| Major Disability | Body composition | **0.66 [0.60, 0.73]** | **<0.001** | **0.66 [0.58, 0.75]** | **<0.001** | **0.70 [0.59, 0.84]** | **<0.001** |
|  | Mental-physical performance | **0.70 [0.64, 0.77]** | **<0.001** | **0.66 [0.57, 0.76]** | **<0.001** | **0.68 [0.58, 0.79]** | **<0.001** |
|  | Muscle strength | **0.84 [0.77, 0.93]** | **<0.001** | **0.80 [0.68, 0.94]** | **0.006** | **0.69 [0.58, 0.81]** | **<0.001** |
|  | Physical activity | **0.83 [0.75, 0.93]** | **<0.001** | **0.82 [0.74, 0.92]** | **<0.001** | 1.02 [0.90, 1.16] | 0.722 |
| All-cause mortality | Body composition | 1.00 [0.88, 1.13] | 0.961 | 0.96 [0.81, 1.12] | 0.586 | **0.76 [0.61, 0.95]** | **0.016** |
|  | Mental-physical performance | **0.65 [0.58, 0.74]** | **<0.001** | **0.54 [0.46, 0.64]** | **<0.001** | **0.60 [0.50, 0.73]** | **<0.001** |
|  | Muscle strength | 1.04 [0.91, 1.17] | 0.585 | 0.82 [0.67, 1.01] | 0.057 | 0.99 [0.80, 1.23] | 0.945 |
|  | Physical activity | **0.79 [0.69, 0.91]** | **<0.001** | **0.78 [0.67, 0.89]** | **<0.001** | **0.78 [0.66, 0.92]** | **0.003** |
| Exceptionally Healthy Aging | Body composition | **1.16 [1.08, 1.25]** | **<0.001** | **1.29 [1.17, 1.41]** | **<0.001** | **1.26 [1.11, 1.43]** | **<0.001** |
|  | Mental-physical performance | **1.26 [1.17, 1.36]** | **<0.001** | **1.31 [1.19, 1.46]** | **<0.001** | **1.29 [****1.15, 1.45]** | **<0.001** |
|  | Muscle strength | 0.97 [0.90, 1.04] | 0.422 | **1.13 [1.01, 1.27]** | **0.033** | **1.22 [1.08, 1.38]** | **0.002** |
|  | Physical activity | **1.17 [1.08, 1.27]** | **<0.001** | **1.18 [1.09, 1.28]** | **<0.001** | 1.05 [0.96, 1.15] | 0.306 |

**Supplementary Table 4. Survival analysis in CARDIA.** Abbreviations: HR, hazard ratio; CI, confidence interval. Adjustments specified in text.

| **Metabolic score** | **Outcome** | **N** | **Events** | **Minimally adjusted** | | **Fully adjusted** | |
| --- | --- | --- | --- | --- | --- | --- | --- |
|  |  |  |  | **HR [95%CI]** | **P value** | **HR [95%CI]** | **P value** |
| Body composition score | Cancer | 2232 | 113 | 0.93 [0.75, 1.15] | 0.49 | 1.07 [0.81, 1.40] | 0.64 |
|  | CVD | 2320 | 125 | 0.77 [0.63, 0.94] | 0.01 | 0.89 [0.69, 1.15] | 0.39 |
|  | Death | 2320 | 155 | 0.77 [0.65, 0.93] | 0.005 | 0.77 [0.61, 0.97] | 0.03 |
| Muscle strength score | Cancer | 2232 | 113 | 0.97 [0.71, 1.33] | 0.84 | 0.90 [0.64, 1.25] | 0.52 |
|  | CVD | 2320 | 125 | 0.97 [0.72, 1.32] | 0.86 | 0.96 [0.68, 1.37] | 0.84 |
|  | Death | 2320 | 155 | 0.57 [0.45, 0.73] | <.0001 | 0.67 [0.50, 0.91] | 0.01 |
| Physical activity score | Cancer | 2232 | 113 | 0.93 [0.77, 1.12] | 0.44 | 0.93 [0.74, 1.16] | 0.52 |
|  | CVD | 2320 | 125 | 0.59 [0.49, 0.71] | <.0001 | 0.71 [0.57, 0.89] | 0.003 |
|  | Death | 2320 | 155 | 0.74 [0.62, 0.87] | 0.0002 | 0.82 [0.68, 1.00] | 0.05 |
| Mental-physical performance score | Cancer | 2232 | 113 | 1.02 [0.77, 1.35] | 0.89 | 0.94 [0.69, 1.27] | 0.68 |
|  | CVD | 2320 | 125 | 0.56 [0.44, 0.72] | <.0001 | 0.64 [0.48, 0.83] | 0.00 |
|  | Death | 2320 | 155 | 0.53 [0.43, 0.66] | <.0001 | 0.70 [0.55, 0.89] | 0.004 |

**Supplementary Figures**

**Supplementary Figure 1. Study Scheme**

**
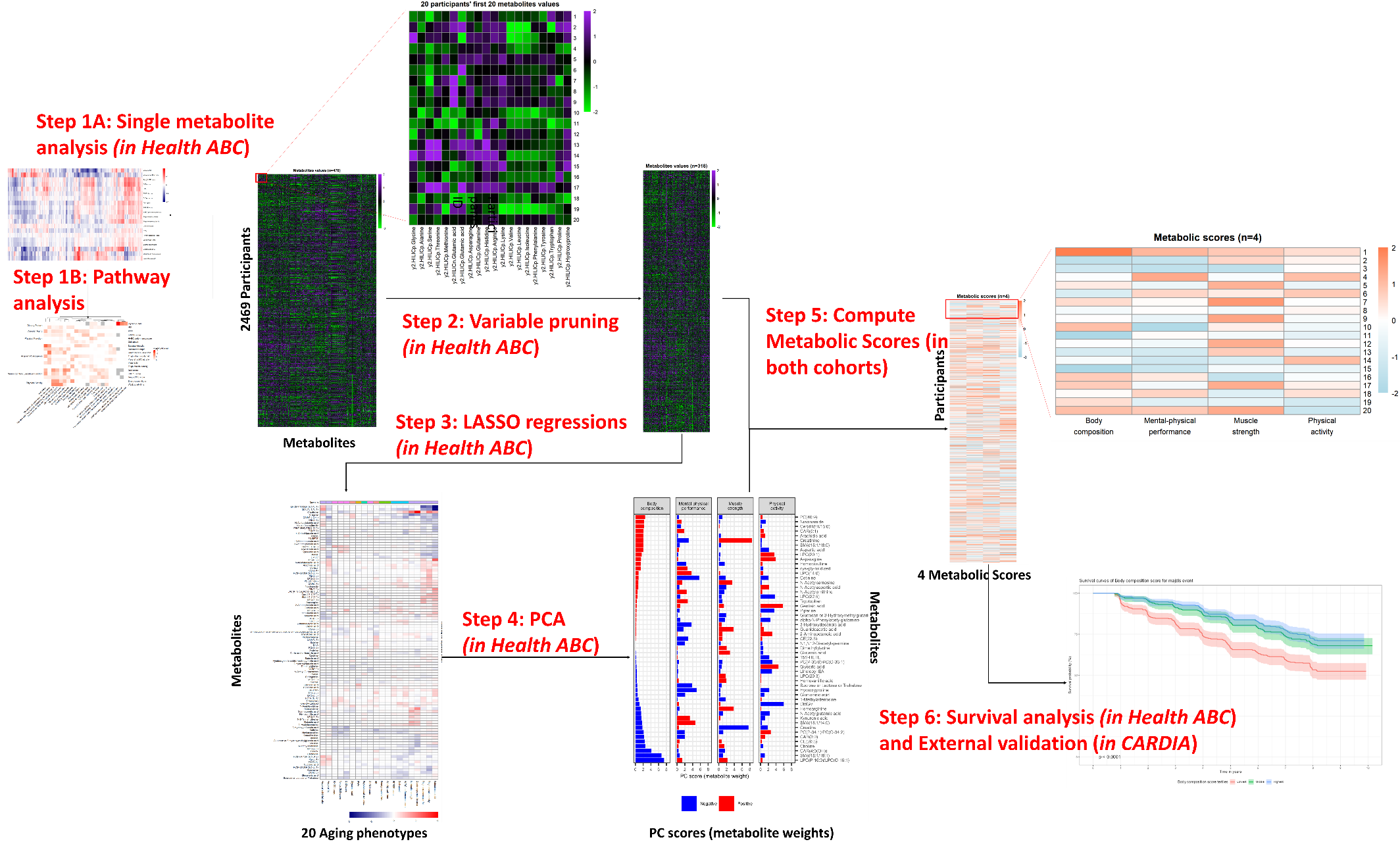
**

**Supplementary Figure 2.** Scree plot of the principal component analysis in Health ABC.


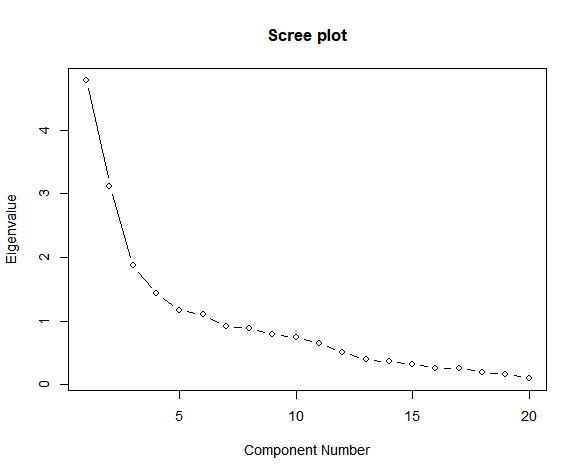


**Supplementary Figure 3.** Distribution of metabolic composite scores across race and sex in Health ABC.


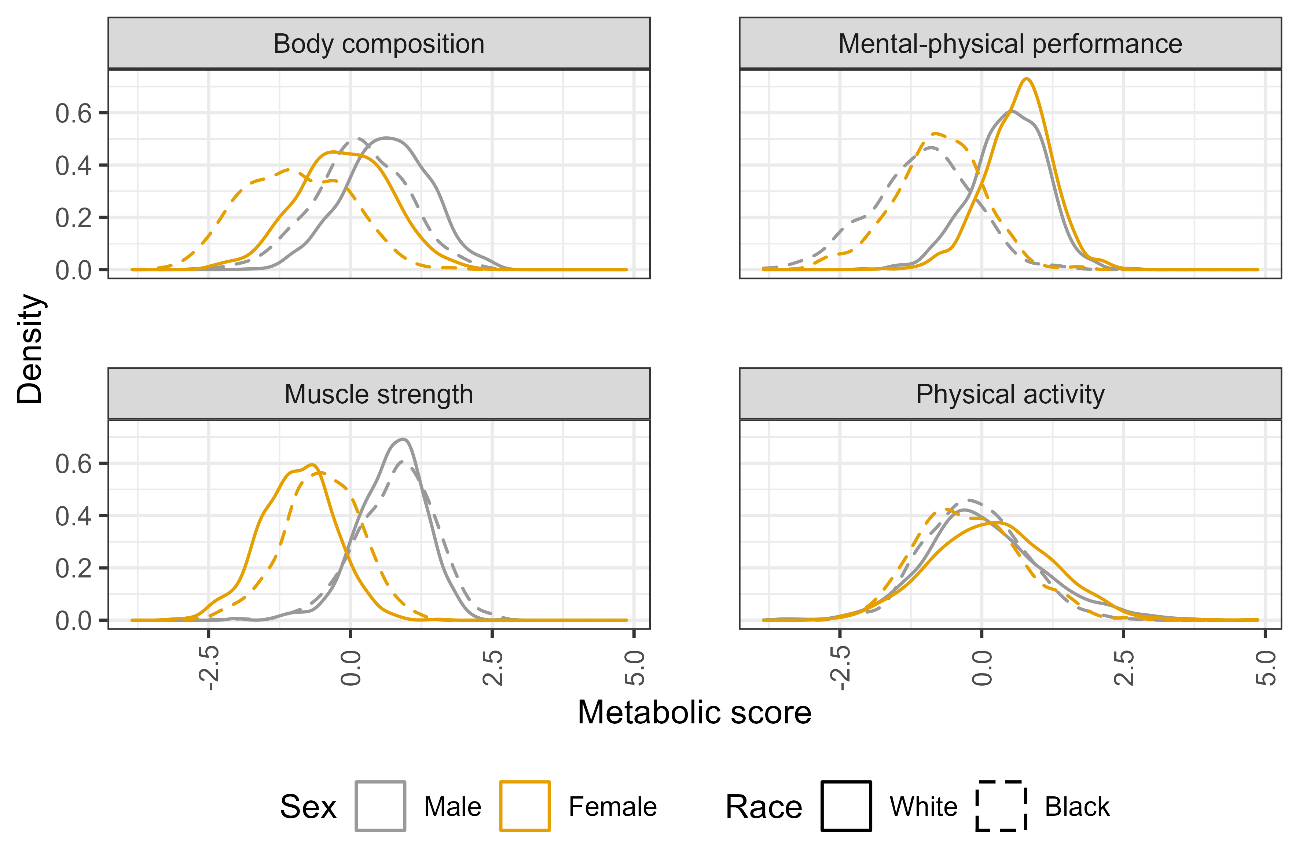


**Supplementary Figure 4.** Distribution of metabolic composite scores across race and sex in CARDIA.


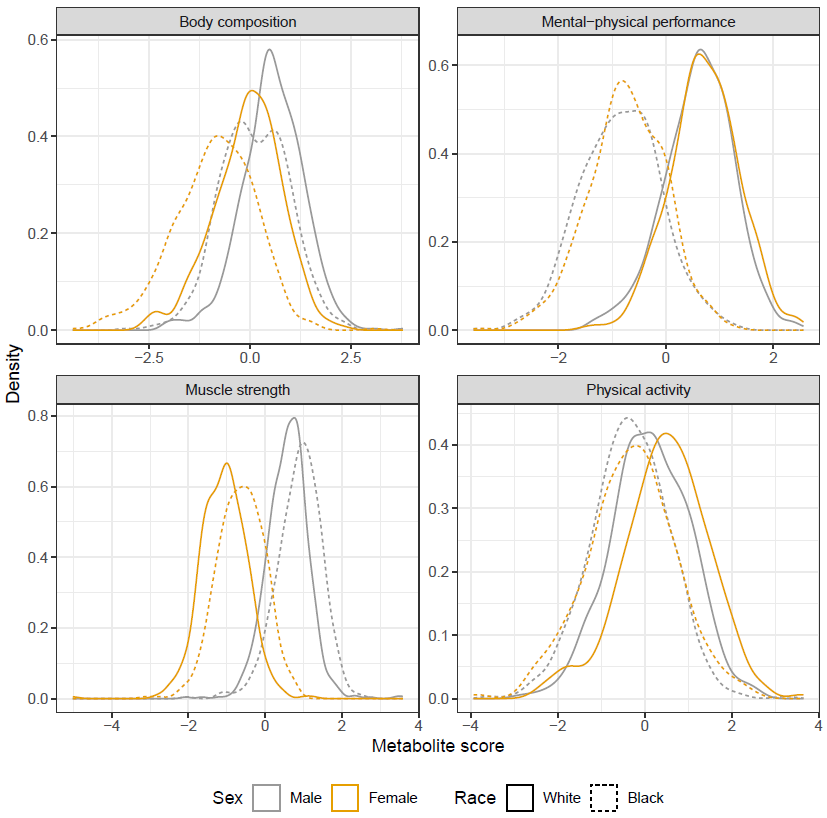


**Excel file of regression estimates (“Online Only”)**

The online-only excel file includes:

- **Linear model regression estimates for each aging phenotype (Health ABC).** Columns include platform (indicating what metabolite platform was used: C18n = C18-negative column; C8p = C8-positive column; HILICp and HILICn = hydrophilic interaction chromatography, negative and positive mode); HMDB ID = human metabolome database identifier (where available); Model description (model adjustments); LASSO beta = LASSO beta coefficient; Single metabolite beta, standard error, P value and FDR = results of linear models
- **Composite score weight (Health ABC).** Each column represents LASSO coefficients for each phenotype (in unadjusted models). Columns labeled “score weights” reflect the weighting (in PCA) of each metabolite in the 4 composite scores (body composition, mental-physical performance, muscle strength, physical activity).
- **Survival analysis (Health ABC).** Cox model results (in adjusted and unadjusted models) for each noted outcome. Beta and SE reflect the Cox model coefficient and its standard error; HR = hazard ratio; Lower CI and Upper CI reflect the upper and lower bounds of the 95% confidence interval.
- **Regression estimates for CARDIA phenotypes.** The two final sheets labeled “Min Adjust” and “Full Adjust” correspond to adjustments in text for all CARDIA aging phenotypes studied. Estimated regression coefficient (beta) and its standard error, P value, and number of observations for the regression (Nobs) is listed.
